# Synthesis, Characterization, and Antifungal Activity of Phenylpyrrole-Substituted Tetramic Acids Bearing Carbonates

**DOI:** 10.3390/molecules21030355

**Published:** 2016-03-21

**Authors:** Wen-Qin Xu, Min Chen, Kun-Yao Wang, Zheng-Jiao Ren, Ai-Min Lu, Chun-Long Yang

**Affiliations:** 1 Jiangsu Key Laboratory of Pesticide Science, Nanjing Agricultural University, Nanjing 210095, China; 2013111008@njau.edu.cn (W.-Q.X.); chenmin@njau.edu.cn (M.C.); 2014111008@njau.edu.cn (K.-Y.W.); 2013111010@njau.edu.cn (Z.-J.R.); 2 Department of Chemistry, College of Science, Nanjing Agricultural University, Nanjing 210095, China; luaimin@njau.edu.cn; 3 Key Laboratory of Monitoring and Management of Crop Diseases and Pest Insects, Ministry of Agriculture, Nanjing Agricultural University, Nanjing 210095, China

**Keywords:** tetramic acid, pyrrole, carbonate, synthesis, crystal structure, antifungal activity

## Abstract

For the aim of discovering new fungicide, a series of phenylpyrrole-substituted tetramic acid derivatives bearing carbonates **6a**–**q** were designed and synthesized via 4-(2,4-dioxopyrrolidin-3-ylidene)-4-(phenylamino)butanoic acids **4a**–**k** and the cyclized products 1′,3,4,5′-tetrahydro-[2,3′-bipyrrolylidene]-2′,4′,5(1*H*)-triones **5a**–**k**. The compounds were characterized using IR, ^1^H- and ^13^C-NMR spectroscopy, mass spectrometry (EI-MS), and elemental analysis. The structure of **6b** was confirmed by X-ray diffraction crystallography. The title compounds **6a**–**q** were bioassayed *in vitro* against the phytopathogenic fungi *Fusarium graminearum*, *Botrytis cinerea* and *Rhizoctonia solani* at a concentration of 100 μg/mL, respectively. Most compounds displayed good inhibitory activity.

## 1. Introduction

Tetramic acid derivatives, which represent an important class of nitrogen-containing heterocycles, have received considerable attention due to their significant biological activities [1], such as antioxidant [2], herbicidal [3,4], phytocytotoxic [5,6,7], anti-HIV [8,9], and antitumor properties [10,11]. Among the abundant bioactivity research of tetramic acid derivatives, 3-heterocycle substituted tetramic acids were proved to be more interesting. Some literatures reported that these compounds showed a wide variety of bioactivity. Fischerellin A, the most active allelochemical compound of *Fischerella muscicola*, exhibited a MIC (Minimal Inhibition Concentration) of 14 nM against *Synechococcus* PPC 6911 and had interesting herbicidal activity [12]. Benzothiadiazine-substituted tetramic acids are potent inhibitors of the hepatitis C virus RNA polymerase [13]. Vermelhotin was obtained from an unidentified fungus CRI247-01, which was found to display cytotoxic activity and antiplasmodial activity with the IC_50_ values of 1–10 μM [14]. Another tetramic acid derivative produced by a plant type-III polyketide synthase showed moderate antiproliferative activity against murine leukemia P388 cells [15]. However, almost no literatures have reported the antifungal activity of 3-heterocycle substituded tetramic acids.

**Scheme 1:**
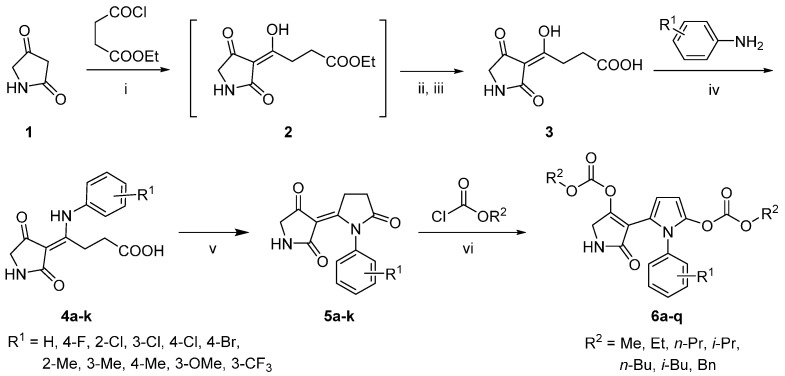
Synthetic route to the title compounds **6a**–**q**. Reaction conditions: (i) 4-Dimethylaminopyridine (DMAP), CH_2_Cl_2_/rt, 10 h; (ii) 10% aq. NaOH/110 °C, 2 h; (iii) 10% aq. HCl (71.7%); (iv) EtOH/90 °C, 6–24 h (56%–90%); (v) DMAP, 1-Ethyl-3-(3-dimethylaminopropyl)carbodiimide hydrochloride (EDCI), CH_2_Cl_2_/rt, 24 h (30.3%–73.1%); (vi) NEt_3_, CHCl_3_/0–5 °C, 0.5–2 h (30.7%–82.4%).

As with many other five-membered heterocyclic compounds, pyrrole derivatives are highly significant in agrichemistry, many of these compounds have been widely used, given their fungicidal [16,17], insecticidal [18,19], and herbicidal [20] activities. In this work, we would like to introduce pyrrole to the 3-position of pyrrolidine-2,4-dione to design and synthesize 17 novel tetramic acid derivatives for revealing the influence of introduced groups (substituted phenylpyrroles and carbonates) on fungicidal activity of pyrrolidine-2,4-diones. Meanwhile, different electronegative and electropositive substitutions, which often incorporated in the structures of commoditized pesticides, were introduced to the phenyl ring to investigate the influence of the substituents and their positions on the antifungal activity. Similarly, in order to investigate whether the types of substituent on carbonate moieties would influence the antifungal activity, eight chloroformates were treated to give the final products. In addition, in view of the continued interest in the development of synthetic routes for preparing heterocyclic systems, an efficient and useful synthesis method of pyrrole was found and reported in this paper (Scheme 1).

## 2. Results and Discussion

### 2.1. Synthesis of the Title Compounds ***6***

Pyrrolidine-2,4-dione **1** was prepared by the literature method [21], starting from ethyl glycinate hydrochloride, through *N*-acylation, Dieckmann cyclization and demethoxycarbonylation. The intermediate 4-(2,4-dioxopyrrolidin-3-ylidene)-4-hydroxybutanoic acid **3** was obtained as a light-yellow powder from compound **1** by successively performing the acylation using ethyl 4-chloro-4-oxobutanoate, saponification with 10% aqueous sodium hydroxide and acidification using 10% aqueous hydrochloride. Compound **3** and different substituted anilines were dissolved in ethanol and refluxed to give 4-(2,4-dioxopyrrolidin-3-ylidene)-4-(substituted phenylamino)butanoic acids **4a**–**k**. Then the synthesis of 1-(substituted phenyl)-1′,3,4,5′-tetrahydro-[2,3′-bipyrrolylidene]-2′,4′,5(1*H*)-triones **5a**–**k** were carried out via intramolecular cyclization of compounds **4** using EDCI as the condensation agent and DMAP as the catalysts. Finally, the title compounds **6a**–**q** were conveniently obtained by the reaction of **5a**–**k** with different chloroformates, respectively. The yields of **6a**–**q** were ranged from 30.7% to 82.4%.

**Scheme 2:**
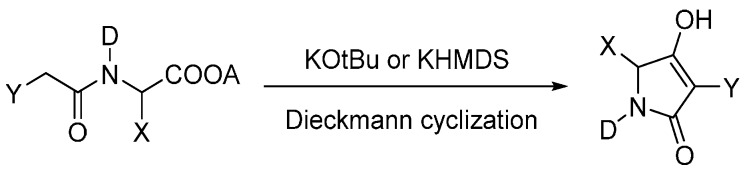
Usual synthesis of 3-aryl or heterocyclic tetramic acid derivatives.

It was reported [22,23] that 3-(aryl or heterocyclic) tetramic acid derivatives were usually generated via Dieckmann cyclization of *N*-(aryl or heterocyclic-acetyl) amino acid esters (Scheme 2). In this paper, pyrroles were formed in the esterification with chloroformates after generation of the pyrrolidine-2-ones, this method was efficient and convenient, which might be useful to synthesize other 3-heterocyclic tetramic acid derivatives.

### 2.2. Tautomerism of the Compounds ***4*** and ***5***

**Scheme 3:**
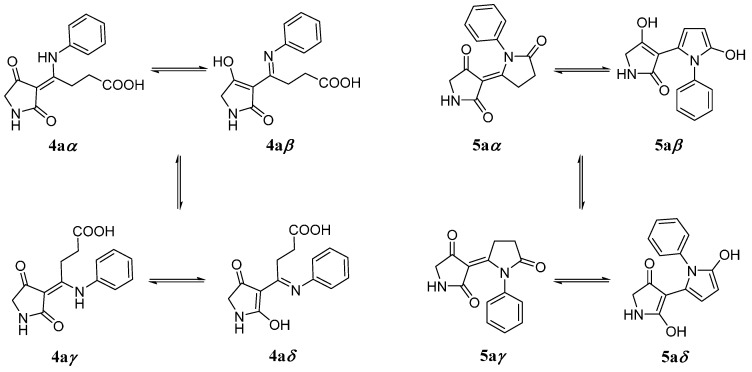
The internal and external tautomerisms of compounds **4** and **5**.

Each compound **4** and **5** possessing a β-tricarbonyl system, can undergo “internal” tautomerism and “external” tautomerism. In order to distinguish the existing forms of the tautomers, the isomers of these compounds were calculated with the Gaussian 03W package [24]. The compounds **4a** and **5a** were chosen as the models for calculation (Scheme 3). The HF/3-21G was used for preliminary optimization, and B3LYP/6-31G* was applied for further optimization. Single point energies of two compounds were calculated with DFT method at B3LYP/6-311++G** level. The solvent effect of DMSO was also taken in account. The calculated results showed that the relative energies of **4aα**–**4aδ** were 0.00, 81.63, 5.12, and 89.09 kJ/mol, while **5aα**–**5a*δ*** were 0.00, 77.8, 0.93, and 99.43 kJ/mol, respectively. This result suggested that **4aα** (*E*-configuration) and **4aγ** (*Z*-configuration) were the stable isomers for compound **4a**, and **4aα** was the major product in the mixture. Similarly, **5aα** (*E*-configuration) and **5aγ** (*Z*-configuration) were the stable isomers for compound **5a**, and **5aα** was the major product. For all products of **4** and **5**, the intensities of the corresponding ^1^H-NMR signals tentatively assigned to the *E* isomers were 70.0% ± 5.0% and 72.5% ± 7.5%, respectively.

### 2.3. X-ray Crystal Structure of Compound ***6b***

The single crystal of compound **6b** was obtained by slow evaporation from the solution composed of chloroform and cyclohexane at room temperature. Diffraction data for this compound were collected with a Bruker Smart APEX II CCD diffractometer (Billerica, MA, USA) with graphite monochromatized Mo Kα radiation (λ = 0.71073 Å) using a φ–ω scan mode at 296 K. The crystal structure was solved and refined by SHELX and SHELXL [25,26]. The crystallographic data are provided in Table 1, Table 2 and Table 3.

**Figure 1:**
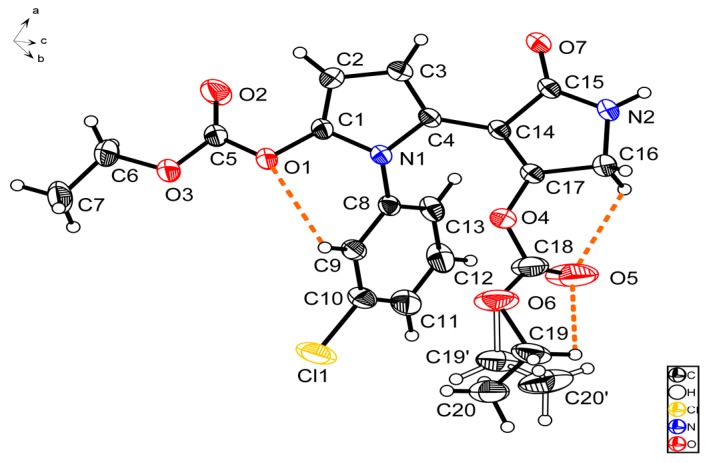
ORTEP diagram of compound **6b** with intramolecular hydrogen bonds.

**Figure 2:**
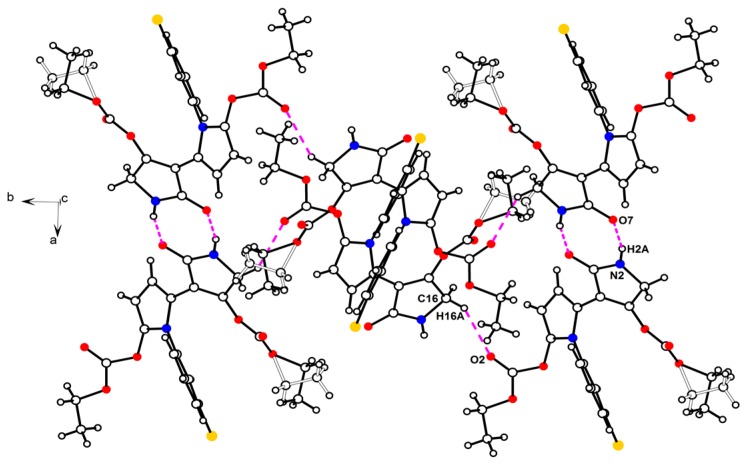
Two-dimensional structure of compound **6b** with intermolecular hydrogen bonds.

**Figure 3:**
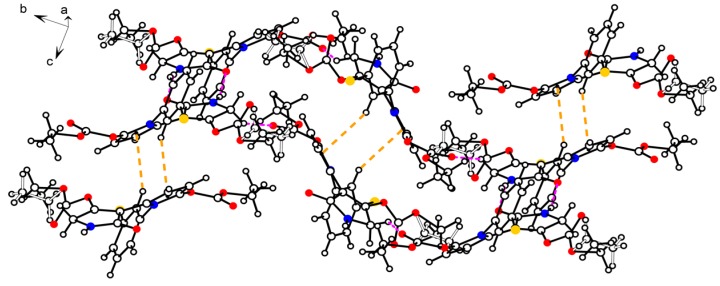
Crystal parking diagram of compound **6b**. Dashed lines show the C-H···π interaction.

In the crystal structure of compound **6b** (Figure 1), the ethyl group connected to the atom O6 appears in a disordered state. In pyrrole system, the bonds C1-N1 and C4-N1 are significantly shorter than the typical single C-N bond and longer than the typical C=N bond, which indicates a significant electron delocalization exists in the pyrrole system. The three rings pyrrole, pyrroline, and benzene are not coplanar, their dihedral angles between pyrrole and pyrrolidone, pyrrole and benzene are 36.379(89)° and 48.522(93)°, respectively. There are three intramolecular hydrogen bonds C9-H9···O1, C19-H19A···O5 and C16-H16B···O5 (Figure 1), which ulteriorly stabilize the molecule. Moreover, other two intermolecular hydrogen bonds N2-H2A···O7 and C16-H16A···O2 between adjacent molecules form a two dimensional chain structure (Figure 2). As shown in Figure 3, C-H···π interaction exists between benzene and pyrrole. The distance between the hydrogen of benzene and the centroid of pyrrole is 3.004 Å. The C-H···π interaction connects the two dimensional chains to form a three-dimensional supramolecular framework.

### 2.4. Antifungal Activity

The inhibition effects of compounds **4**–**6** were tested *in vitro* against the phytopathogenic fungi *Fusarium graminearum*, *Botrytis cinerea,* and *Rhizoctonia solani* using mycelium growth rate method at the concentration of 100 μg/mL [27]. Compounds **4** and **5** were almost inactive against all tested fungus, such as **4a** and **5a**, their inhibition rates were less than 20%, while the title compounds of **6** exihibited obvious antifungal activities.

As shown in Table 4, *B. cinerea* and *R. solani* were more sensitive than *F. graminearaum* to most members of the compounds. Compound **6h** showed the highest activity with an inhibitory rate of 82.2% against *B. cinerea*. It can be noticed that compounds **6d** and **6f** carrying two *i*-propyl or *i*-butyl groups displayed relatively better antifungal activity against the three kinds of fungi than compounds **6a**–**c**, **6e**, and **6g** carrying two methyl, ethyl, *n*-propyl, *n*-butyl, or benzyl groups did. Screening data of compounds **6a**–**6g** indicated that introducing medium-sized alkyls to the carbonate moiety may elevate the antifungal activity. Meanwhile, there is no direct relationship between antifungal activities and substituents of phenyl ring compared with the antifungal activities of compounds **6f** and **6h**–**6q**. That is, neither electronegative nor electropositive substitutions at phenyl have played a crucial role in the activity.

## 3. Experimental Section

### 3.1. General

All melting points of the title compounds were determined on an uncorrected WRS-1B digital melting point apparatus. IR spectra (4000–400 cm^–1^) were recorded on a Bruker Tensor 27 FT-IR spectrometer, using KBr disks. ^1^H-NMR and ^13^C-NMR spectra were measured on Bruker 400 spectrometer (DMSO-*d*_6_ or CDCl_3_ as solvent, TMS as internal standard). Mass spectra were recorded on a TRACE 2000 spectrometer. The elemental analyses were performed on an Elementar Vario EL cube analyzer. CCDC-1432180 contains the supplementary crystallographic data for this paper. These data can be obtained free of charge via http://www.ccdc.cam.ac.uk/conts/retrieving.html (or from the CCDC, 12 Union Road, Cambridge CB2 1EZ, UK; Fax: +44-1223-336033; E-mail: deposit@ccdc.cam.ac.uk). Progress of the reactions was monitored by thin layer chromatography (TLC). All reagents and solvents were obtained from commercial suppliers. Reagents were analytically or chemically pure and were not further purified. All the solvents were dried by standard methods in advance.

### 3.2. General Procedure for the Synthesis of Compounds ***3***

A mixture of DMAP (14.2 g, 116.2 mmol) in dichloromethane (50 mL) was added to a mixture of pyrrolidine-2,4-dione **1** (5.0 g, 50.5 mmol) and ethyl succinyl chloride (8.3 g, 50.5 mmol) in dichloromethane (100 mL) at 0 °C. The resulting mixture was stirred at 25 °C for 10 h. Then the mixture was washed successively with 10% aqueous HCl, saturated brine and water, dried with Na_2_SO_4_, filtered and concentrated *in vacuo* to yield the crude product **2** (9.5 g) as a yellow solid which was used directly in the next step.

10% aqueous NaOH (100 mL) was added to the above obtained product **2** (9.5 g). The resulting mixture was stirred at 110 °C for 2 h. Then the mixture was allowed to cool to room temperature, acidified with 10% aqueous HCl to pH = 2–3 and precipitated. The yellow solid was collected by filtration, rinsed with water, and dried in the air to afford the desired product **3** (7.2 g, 36.2 mmol) with yield of 71.7%, m.p. 198.8 °C (decomp). ^1^H-NMR (DMSO-*d*_6_) δ (ppm): 12.24 (s, 1H, COOH), 9.73 (s, 1H, C=COH), 8.80 (s, 1H, NH), 3.80 (s, 2H, C*H*_2_NH), 3.01 (t, *J* = 6.9 Hz, 2H, C=CCH_2_), 2.53 (t, *J* = 7.0 Hz, 2H, C*H*_2_COOH); EI-MS (*m*/*z*) 199.1 [M]^+^.

### 3.3. General Procedure for the Synthesis of Compounds ***4***

A mixture of compound **3** (1.5 g, 7.5 mmol) and a substituted aniline (7.5 mmol) was refluxed in ethanol (25 mL) for 6–24 h. After cooling, the resulting solid product was collected by filtration and recrystallized from EtOH to give the desired products **4**.

*4-(2,4-Dioxopyrrolidin-3-ylidene)-4-(phenylamino)butanoic acid* (**4a**). White solid (76.3%), m.p. 218.4 °C (decomp). IR (KBr, cm^−1^) ν: 3314, 3070, 2930, 1724, 1666, 1567, 1438, 1272, 1189, 1080, 896, 787; ^1^H-NMR (400 MHz, DMSO-*d*_6_) δ (ppm): 12.33 (s, (0.3)1H (*Z*), PhNH), 12.31 (s, 1H, COOH), 12.26 (s, (0.7)1H (*E*), PhNH), 7.92 (s, (0.7)1H (*E*), CH_2_N*H*), 7.52 (s, (0.3)1H (*Z*), CH_2_N*H*), 7.48 (d, *J* = 7.4 Hz, 2H, PhH), 7.44–7.31 (m, 3H, PhH), 3.70 (s, (0.6)2H (*Z*), C*H*_2_NH), 3.61 (s, (1.4)2H (*E*), C*H*_2_NH), 3.06–2.98 (m, (0.6)2H (*Z*), C=CCH_2_), 2.98–2.89 (m, (1.4)2H (*E*), C=CCH_2_), 2.49–2.42 (m, 2H, C*H*_2_COOH); ^13^C-NMR (101 MHz, DMSO-*d*_6_) δ (ppm): 198.32 (*Z*), 193.77 (*E*), 175.63 (*E*), 173.21 (*E*), 173.12 (*Z*), 171.59 (*Z*), 168.71 (*Z*), 168.35 (*E*), 136.33 (*E*), 136.27 (*Z*), 130.07 (2 × C), 128.34 (*Z*), 128.11 (*E*), 126.51 (2 × C (*Z*)), 126.23 (2 × C (*E*)), 97.98 (*Z*), 96.11 (*E*), 50.82 (*E*), 49.59 (*Z*), 32.08 (*Z*), 31.45 (*E*), 23.64 (*E*), 22.85 (*Z*); EI-MS (*m*/*z*) 274.1 [M]^+^. Anal. Calcd. for C_1__4_H_14_N_2_O_4_: C, 61.31; H, 5.14; N, 10.21. Found: C, 61.44; H, 5.25; N, 10.07.

*4-(2,4-Dioxopyrrolidin-3-ylidene)-4-((4-fluorophenyl)amino)butanoic acid* (**4b**). White solid (80.3%), m.p. 223.8 °C (decomp). IR (KBr, cm^−1^) ν: 3313, 3070, 2885, 1723, 1667, 1568, 1484, 1379, 1271, 1187, 1084, 803, 741; ^1^H-NMR (400 MHz, DMSO-*d*_6_) δ (ppm): 12.30 (s, 1H, COOH), 12.19 (s, (0.3)1H (*Z*), PhNH), 12.12 (s, (0.7)1H (*E*), PhNH), 7.90 (s, (0.7)1H (*E*), CH_2_N*H*), 7.51 (s, (0.3)1H (*Z*), CH_2_N*H*), 7.48–7.38 (m, 2H, PhH), 7.44–7.31 (m, 2H, PhH), 3.70 (s, (0.6)2H (*Z*), C*H*_2_NH), 3.61 (s, (1.4)2H (*E*), C*H*_2_NH), 3.01–2.94 (m, (0.6)2H (*Z*), C=CCH_2_), 2.94–2.86 (m, (1.4)2H (*E*), C=CCH_2_), 2.49–2.42 (m, 2H, CH_2_COOH); ^13^C-NMR (101 MHz, DMSO-*d*_6_) δ (ppm): 198.30 (*Z*), 193.79 (*E*), 175.56 (*E*), 173.20 (*E*), 173.12 (*Z*), 171.57 (*Z*), 169.01 (*Z*), 168.68 (*E*), 161.54 (d, *J*_CF_ = 245.1 Hz), 132.66 (d, *J*_CF_ = 2.9 Hz), 129.09 (d, *J*_CF_ = 9.0 Hz, 2 × C (*Z*)), 128.82 (d, *J*_CF_ = 8.8 Hz, 2 × C (*E*)), 116.81 (d, *J*_CF_ = 22.8 Hz, 2 × C), 97.98 (*Z*), 96.13 (*E*), 50.85 (*E*), 49.59 (*Z*), 32.04 (*Z*), 31.41 (*E*), 23.59 (*E*), 22.81 (*Z*); EI-MS (*m*/*z*) 292.1 [M]^+^. Anal. Calcd. for C_14_H_13_FN_2_O_4_: C, 57.53; H, 4.48; N, 9.59. Found: C, 57.64; H, 4.62; N, 9.47.

*4-((2-Chlorophenyl)amino)-4-(2,4-dioxopyrrolidin-3-ylidene)butanoic acid* (**4c**). White solid (56.0%), m.p. 246.2 °C (decomp). IR (KBr, cm^−1^) ν: 3171, 3040, 2912, 1719, 1676, 1640, 1566, 1467, 1376, 1254, 1199, 1056, 988, 756; ^1^H-NMR (400 MHz, DMSO-*d*_6_) δ (ppm): 12.32 (s, 1H, COOH), 12.18 (s, 1H, PhNH), 7.99 (s, (0.7)1H (*E*), CH_2_N*H*), 7.71–7.64 (m, 1H, PhH), 7.64–7.58 (m, 1H, PhH), 7.57 (s, (0.3)1H (*Z*), CH_2_N*H*), 7.50–7.40 (m, 2H, PhH), 3.74 (s, (0.6)2H (*Z*), C*H*_2_NH), 3.64 (s, (1.4)2H (*E*), C*H*_2_NH), 2.98–2.92 (m, (0.6)2H (*Z*), C=CCH_2_), 2.92–2.83 (m, (1.4)2H (*E*), C=CCH_2_), 2.45–2.35 (m, 2H, C*H*_2_COOH); ^13^C-NMR (101 MHz, DMSO-*d*_6_) δ (ppm): 198.76 (*Z*), 194.01 (*E*), 175.30 (*E*), 173.04 (*E*), 172.95 (*Z*), 171.25 (*Z*), 168.84 (*Z*), 168.54 (*E*), 133.87 (*E*), 133.84 (*Z*), 130.58, 130.50 (*Z*), 130.45 (*E*), 130.25 (*Z*), 130.00 (*E*), 129.55 (*Z*), 129.22 (*E*), 128.69, 98.61 (*Z*), 96.85 (*E*), 50.94 (*E*), 49.64 (*Z*), 31.94 (*Z*), 31.33 (*E*), 23.67 (*E*), 22.92 (*Z*); EI-MS (*m*/*z*) 308.1 [M]^+^. Anal. Calcd. for C_1__4_H_1__3_ClN_2_O_4_: C, 54.47; H, 4.24; N, 9.07. Found: C, 54.36; H, 4.35; N, 9.18.

*4-((3-Chlorophenyl)amino)-4-(2,4-dioxopyrrolidin-3-ylidene)butanoic acid* (**4d**). White solid (70.6%), m.p. 223.5 °C (decomp). IR (KBr, cm^−1^) ν: 3169, 3037, 2923, 1715, 1644, 1560, 1470, 1342, 1255, 1159, 867, 713; ^1^H-NMR (400 MHz, DMSO-*d*_6_) δ (ppm): 12.31 (s, 1H, COOH), 12.24 (s, 1H, PhNH), 7.96 (s, (0.7)1H (*E*), CH_2_N*H*), 7.58–7.45 (m, 3H, PhH), 7.44(s, (0.3)1H (*Z*), CH_2_N*H*), 7.35 (t, *J* = 9.9 Hz, 1H, PhH), 3.71 (s, (0.6)2H (*Z*), C*H*_2_NH), 3.62 (s, (1.4)2H (*E*), C*H*_2_NH), 3.05–2.99 (m, (0.6)2H (*Z*), C=CCH_2_), 2.98–2.90 (m, (1.4)2H (*E*), C=CCH_2_), 2.49–2.43 (m, 2H, C*H*_2_COOH); ^13^C-NMR (101 MHz, DMSO-*d*_6_) δ (ppm): 198.41 (*Z*), 193.93 (*E*), 175.36 (*E*), 173.21 (*E*), 173.13 (*Z*), 171.40 (*Z*), 168.50 (*Z*), 168.22 (*E*), 137.99 (*E*), 137.92 (*Z*), 134.13 (*E*), 134.10 (*Z*), 131.50 (*E*), 131.47 (*Z*), 128.26 (*Z*), 128.00 (*E*), 126.64 (*Z*), 126.29 (*E*), 125.53 (*Z*), 125.19 (*E*), 98.38 (*Z*), 96.59 (*E*), 50.85 (*E*), 49.61 (*Z*), 32.07 (*Z*), 31.42 (*E*), 23.63 (*E*), 22.88 (*Z*); EI-MS (*m*/*z*) 308.1 [M]^+^. Anal. Calcd. for C_1__4_H_1__3_ClN_2_O_4_: C, 54.47; H, 4.24; N, 9.07. Found: C, 54.56; H, 4.14; N, 8.93.

*4-((4-Chlorophenyl)amino)-4-(2,4-dioxopyrrolidin-3-ylidene)butanoic acid* (**4e**). White solid (70.0%), m.p. 230.5 °C (decomp). IR (KBr, cm^−1^) ν: 3322, 3065, 2926, 1709, 1672, 1560, 1482, 1378, 1246, 1093, 807, 715; ^1^H-NMR (400 MHz, DMSO-*d*_6_) δ (ppm): 12.30 (s, 1H, COOH), 12.23 (s, (0.3)1H (*Z*), PhNH), 12.20 (s, (0.7)1H (*E*), PhNH), 7.93 (s, (0.7)1H (*E*), CH_2_N*H*), 7.55 (s, (0.3)1H (*Z*), CH_2_N*H*), 7.53 (d, *J* = 8.4 Hz, 2H, PhH), 7.40 (t, *J* = 10.1 Hz, 2H, PhH), 3.70 (s, (0.6)2H (*Z*), C*H*_2_NH), 3.61 (s, (1.4)2H (*E*), C*H*_2_NH), 3.05–2.97 (m, (0.6)2H (*Z*), C=CCH_2_), 2.97–2.89 (m, (1.4)2H (*E*), C=CCH_2_), 2.48–2.42 (m, 2H, C*H*_2_COOH); ^13^C-NMR (101 MHz, DMSO-*d*_6_) δ (ppm): 198.37 (*Z*), 193.85 (*E*), 175.45 (*E*), 173.18 (*E*), 173.10 (*Z*), 171.46 (*Z*), 168.61 (*Z*), 168.30 (*E*), 135.41 (*E*), 135.36 (*Z*), 132.71 (*Z*), 132.45 (*E*), 129.95 (2 × C), 128.53 (2 × C (*Z*)), 128.21 (2 × C (*E*)), 98.26 (*Z*), 96.44 (*E*), 50.84 (*E*), 49.60 (*Z*), 32.08 (*Z*), 31.44 (*E*), 23.60 (*E*), 22.83 (*Z*); EI-MS (*m*/*z*) 308.1 [M]^+^. Anal. Calcd. for C_1__4_H_1__3_ClN_2_O_4_: C, 54.47; H, 4.24; N, 9.07. Found: C, 54.39; H, 4.10; N, 9.22.

*4-((4-Bromophenyl)amino)-4-(2,4-dioxopyrrolidin-3-ylidene)butanoic acid* (**4f**). White solid (83.6%), m.p. 239.8 °C (decomp). IR (KBr, cm^−1^) ν: 3327, 3065, 2952, 1709, 1671, 1559, 1400, 1377, 1247, 1072, 805, 713; ^1^H-NMR (400 MHz, DMSO-*d*_6_) δ (ppm): 12.31 (s, 1H, COOH), 12.21 (s, (0.3)1H (*Z*), PhNH), 12.19 (s, (0.7)1H (*E*), PhNH), 7.94 (s, (0.7)1H (*E*), CH_2_N*H*), 7.71–7.63 (m, 2H, PhH), 7.54 (s, (0.3)1H (*Z*), CH_2_N*H*), 7.33 (t, *J* = 10.0 Hz, 2H, PhH), 3.70 (s, (0.6)2H (*Z*), C*H*_2_NH), 3.61 (s, (1.4)2H (*E*), C*H*_2_NH), 3.05–2.97 (m, (0.6)2H (*Z*), C=CCH_2_), 2.97–2.89 (m, (1.4)2H (*E*), C=CCH_2_), 2.49–2.41 (m, 2H, C*H*_2_COOH); ^13^C-NMR (101 MHz, DMSO-*d*_6_) δ (ppm): 198.38 (*Z*), 193.85 (*E*), 175.44 (*E*), 173.19 (*E*), 173.11 (*Z*), 171.44 (*Z*), 168.51 (*Z*), 168.20 (*E*), 135.86 (*E*), 135.80 (*Z*), 132.88 (2 × C), 128.79 (2 × C (*Z*)), 128.47 (2 × C (*E*)), 121.11 (*Z*), 120.82 (*E*), 98.30 (*Z*), 96.48 (*E*), 50.84 (*E*), 49.61 (*Z*), 32.10 (*Z*), 31.45 (*E*), 23.60 (*E*), 22.84 (*Z*); EI-MS (*m*/*z*) 352.0 [M]^+^. Anal. Calcd. for C_1__4_H_1__3_BrN_2_O_4_: C, 47.61; H, 3.71; N, 7.93. Found: C, 47.68; H, 3.85; N, 8.06.

*4-(2,4-Dioxopyrrolidin-3-ylidene)-4-(o-tolylamino)butanoic acid* (**4g**). White solid (67.7%), m.p. 235.4 °C (decomp). IR (KBr, cm^−1^) ν: 3180, 3041, 2914, 1724, 1643, 1560, 1379, 1253, 1200, 1162, 849, 753; ^1^H-NMR (400 MHz, DMSO-*d*_6_) δ (ppm): 12.28 (s, 1H, COOH), 12.22 (s, (0.3)1H (*Z*), PhNH), 12.14 (s, (0.7)1H (*E*), PhNH), 7.88 (s, (0.7)1H (*E*), CH_2_N*H*), 7.50 (s, (0.3)1H (*Z*), CH_2_N*H*), 7.38 (d, *J* = 3.9 Hz, 1H, PhH), 7.35–7.26 (m, 3H, PhH), 3.71 (s, (0.6)2H (*Z*), C*H*_2_NH), 3.61 (s, (1.4)2H (*E*), C*H*_2_NH), 2.96–2.90 (m, (0.6)2H (*Z*), C=CCH_2_), 2.89–2.83 (m, (1.4)2H (*E*), C=CCH_2_), 2.46–2.35 (m, 2H, C*H*_2_COOH), 2.22 (s, 3H, CH_3_); ^13^C-NMR (101 MHz, DMSO-*d*_6_) δ (ppm): 198.45 (*Z*), 193.64 (*E*), 175.82 (*E*), 173.18 (*E*), 173.09 (*Z*), 171.66 (*Z*), 169.25 (*Z*), 168.88 (*E*), 135.20, 134.42 (*Z*), 134.39 (*E*), 131.45, 128.75 (*Z*), 128.52 (*E*), 127.45 (*Z*), 127.35 (*Z*), 127.32 (*E*), 127.16 (*E*), 98.00 (*Z*), 96.09 (*E*), 50.86 (*E*), 49.59 (*Z*), 31.92 (*Z*), 31.32 (*E*), 23.64 (*E*), 22.90 (*Z*), 17.94 (*E*), 17.92 (*Z*); EI-MS (*m*/*z*) 288.1 [M]^+^. Anal. Calcd. for C_1__5_H_1__6_N_2_O_4_: C, 62.49; H, 5.59; N, 9.72. Found: C, 62.41; H, 5.47; N, 9.86.

*4-(2,4-Dioxopyrrolidin-3-ylidene)-4-(m-tolylamino)butanoic acid* (**4h**). White solid (73.7%), m.p. 214.7 °C (decomp). IR (KBr, cm^−1^) ν: 3172, 3038, 2923, 1721, 1644, 1568, 1442, 1376, 1260, 1163, 1037, 878, 706; ^1^H-NMR (400 MHz, DMSO-*d*_6_) δ (ppm): 12.34 (s, 1H, COOH), 12.32 (s, (0.3)1H (*Z*), PhNH), 12.24 (s, (0.7)1H (*E*), PhNH), 7.91 (s, (0.7)1H (*E*), CH_2_N*H*), 7.52 (s, (0.3)1H (*Z*), CH_2_N*H*), 7.36 (t, *J* = 7.5 Hz, 1H, PhH), 7.25–7.10 (m, 3H, PhH), 3.70 (s, (0.6)2H (*Z*), C*H*_2_NH), 3.60 (s, (1.4)2H (*E*), C*H*_2_NH), 3.05–2.98 (m, (0.6)2H (*Z*), C=CCH_2_), 2.98–2.90 (m, (1.4)2H (*E*), C=CCH_2_), 2.49–2.42 (m, 2H, C*H*_2_COOH), 2.34 (s, 3H, CH_3_); ^13^C-NMR (101 MHz, DMSO-*d*_6_) δ (ppm): 198.30 (*Z*), 193.73 (*E*), 175.66 (*E*), 173.23 (*E*), 173.14 (*Z*), 171.61 (*Z*), 168.70 (*Z*), 168.32 (*E*), 139.77, 136.23 (*E*), 136.15 (*Z*), 129.83, 128.96 (*Z*), 128.74 (*E*), 126.79 (*Z*), 126.53 (*E*), 123.39 (*Z*), 123.10 (*E*), 97.94 (*Z*), 96.05 (*E*), 50.81 (*E*), 49.59 (*Z*), 32.14 (*Z*), 31.50 (*E*), 23.67 (*E*), 22.89 (*Z*), 21.25; EI-MS (*m*/*z*) 288.1 [M]^+^. Anal. Calcd. for C_1__5_H_1__6_N_2_O_4_: C, 62.49; H, 5.59; N, 9.72. Found: C, 62.40; H, 5.71; N, 9.84.

*4-(2,4-Dioxopyrrolidin-3-ylidene)-4-(p-tolylamino)butanoic acid* (**4i**). White solid (90.0%), m.p. 234.2 °C (decomp). IR (KBr, cm^−1^) ν: 3302, 3034, 2924, 1725, 1665, 1590, 1441, 1384, 1247, 1162, 1084, 820, 706; ^1^H-NMR (400 MHz, DMSO-*d*_6_) δ (ppm): 12.32 (s, 1H, COOH), 12.27 (s, (0.3)1H (*Z*), PhNH), 12.17 (s, (0.7)1H (*E*), PhNH), 7.86 (s, (0.7)1H (*E*), CH_2_N*H*), 7.47 (s, (0.3)1H (*Z*), CH_2_N*H*), 7.28 (d, *J* = 7.9 Hz, 2H, PhH), 7.23 (t, *J* = 9.3 Hz, 2H, PhH), 3.69 (s, (0.6)2H (*Z*), C*H*_2_NH), 3.59 (s, (1.4)2H (*E*), C*H*_2_NH), 3.03–2.95 (m, (0.6)2H (*Z*), C=CCH_2_), 2.95–2.88 (m, (1.4)2H (*E*), C=CCH_2_), 2.49–2.41 (m, 2H, C*H*_2_COOH), 2.34 (s, 3H, CH_3_); ^13^C-NMR (101 MHz, DMSO-*d*_6_) δ (ppm): 198.24 (*Z*), 193.68 (*E*), 175.70 (*E*), 173.21 (*E*), 173.13 (*Z*), 171.65 (*Z*), 168.88 (*Z*), 168.50 (*E*), 137.90 (*Z*), 137.67 (*E*), 133.67 (*E*), 133.62 (*Z*), 130.52 (2 × C), 126.33 (2 × C (*Z*)), 126.11 (2 × C (*E*)), 97.83 (*Z*), 95.94 (*E*), 50.82 (*E*), 49.59 (*Z*), 32.09 (*Z*), 31.46 (*E*), 23.63 (*E*), 22.84 (*Z*), 21.05; EI-MS (*m*/*z*) 288.1 [M]^+^. Anal. Calcd. for C_1__5_H_1__6_N_2_O_4_: C, 62.49; H, 5.59; N, 9.72. Found: C, 62.63; H, 5.72; N, 9.63.

*4-(2,4-Dioxopyrrolidin-3-ylidene)-4-((3-methoxyphenyl)amino)butanoic acid* (**4j**). White solid (79.9%), m.p. 202.6 °C (decomp). IR (KBr, cm^−1^) ν: 3315, 3070, 2945, 1728, 1666, 1560, 1454, 1379, 1268, 1151, 1043, 869, 785; ^1^H-NMR (400 MHz, DMSO-*d*_6_) δ (ppm): 12.31 (s, 1H, COOH), 12.30 (s, (0.3)1H (*Z*), PhNH), 12.23 (s, (0.7)1H (*E*), PhNH), 7.91 (s, (0.7)1H (*E*), CH_2_N*H*), 7.51 (s, (0.3)1H (*Z*), CH_2_N*H*), 7.42–7.35 (m, 1H, PhH), 7.01–6.87 (m, 3H, PhH), 3.78 (s, 3H, CH_3_), 3.70 (s, (0.6)2H (*Z*), C*H*_2_NH), 3.60 (s, (1.4)2H (*E*), C*H*_2_NH), 3.06–3.00 (m, (0.6)2H (*Z*), C=CCH_2_), 3.00–2.93 (m, (1.4)2H (*E*), C=CCH_2_), 2.54–2.45 (m, 2H, C*H*_2_COOH); ^13^C-NMR (101 MHz, DMSO-*d*_6_) δ (ppm): 198.32 (*Z*), 193.78 (*E*), 175.60 (*E*), 173.25 (*E*), 173.17 (*Z*), 171.57 (*Z*), 168.72 (*Z*), 168.35 (*E*), 160.45 (*E*), 160.43 (*Z*), 137.48 (*E*), 137.39 (*Z*), 130.84, 118.48 (*Z*), 118.22 (*E*), 114.25 (*Z*), 114.03 (*E*), 111.97 (*Z*), 111.67 (*E*), 98.02 (*Z*), 96.15 (*E*), 55.87 (*Z*), 55.85 (*E*), 50.83 (*E*), 49.59 (*Z*), 32.17 (*Z*), 31.52 (*E*), 23.75 (*E*), 22.96 (*Z*); EI-MS (*m*/*z*) 304.1 [M]^+^. Anal. Calcd. for C_1__5_H_1__6_N_2_O_5_: C, 59.21; H, 5.30; N, 9.21. Found: C, 59.06; H, 5.18; N, 9.13.

*4-(2,4-Dioxopyrrolidin-3-ylidene)-4-((3-(trifluoromethyl)phenyl)amino)butanoic acid* (**4k**). White solid (69.8%), m.p. 215.9 °C (decomp). IR (KBr, cm^−1^) ν: 3300, 3070, 2941, 1719, 1681, 1569, 1431, 1322, 1254, 1116, 991, 797; ^1^H-NMR (400 MHz, DMSO-*d*_6_) δ (ppm): 12.31 (s, 1H, COOH), 12.29 (s, 1H, PhNH), 7.98 (s, (0.7)1H (*E*), CH_2_N*H*), 7.82–7.65 (m, 4H, PhH), 7.58 (s, (0.3)1H (*Z*), CH_2_N*H*), 3.72 (s, (0.6)2H (*Z*), C*H*_2_NH), 3.63 (s, (1.4)2H (*E*), C*H*_2_NH), 3.03–2.97 (m, (0.6)2H (*Z*), C=CCH_2_), 2.97–2.91 (m, (1.4)2H (*E*), C=CCH_2_), 2.55–2.44 (m, 2H, C*H*_2_COOH); ^13^C-NMR (101 MHz, DMSO-*d*_6_) δ (ppm): 198.40 (*Z*), 193.98 (*E*), 175.31 (*E*), 173.17 (*E*), 173.10 (*Z*), 171.38 (*Z*), 168.49 (*Z*), 168.26 (*E*), 137.45 (*E*), 137.41 (*Z*), 131.17 (*E*), 131.12 (*Z*), 130.92 (*Z*), 130.63 (q, *J*_CF_ = 32.2 Hz), 130.55 (*E*), 124.55 (q, *J*_CF_ = 3.6 Hz), 124.12 (q, *J*_CF_ = 272.6 Hz), 123.23 (q, *J*_CF_ = 3.7 Hz), 98.52 (*Z*), 96.76 (*E*), 50.86 (*E*), 49.63 (*Z*), 32.07 (*Z*), 31.40 (*E*), 23.67 (*E*), 22.95 (*Z*); EI-MS (*m*/*z*) 342.1 [M]^+^. Anal. Calcd. for C_15_H_13_F_3_N_2_O_4_: C, 52.64; H, 3.83; N, 8.18. Found: C, 52.48; H, 3.93; N, 8.04.

### 3.4. General Procedure for the Synthesis of Compounds ***5***

A mixture of compound **4** (4 mmol), EDCI (4.8 mmol) and DMAP (4.6 mmol) in dichloromethane (30 mL) was stirred at room temperature for 24 h. The resulting solid product was collected by filtration and recrystallized from MeOH to give the desired products **5**.

*1-Phenyl-1′,3,4,5′-tetrahydro-[2,3′-bipyrrolylidene]-2′,4′,5(1H)-trione* (**5a**). White solid (71.4%), m.p. 257.5 °C (decomp). IR (KBr, cm^−1^) ν: 3199, 3063, 2899, 1752, 1713, 1667, 1556, 1453, 1353, 1289, 1106, 853, 758; ^1^H-NMR (400 MHz, DMSO-*d*_6_) δ (ppm): 7.92 (s, (0.65)1H (*E*), NH), 7.39 (s, (0.35)1H (*Z*), NH), 7.39–7.25 (m, 3H, PhH), 7.21–7.13 (m, 2H, PhH), 3.63 (s, (0.7)2H (*Z*), C*H*_2_NH), 3.53–3.47 (m, (1.3)2H (*E*), C=CCH_2_), 3.46–3.40 (m, (0.7)2H (*Z*), C=CCH_2_), 3.37 (s, (1.3)2H (*E*), C*H*_2_NH), 2.79–2.69 (m, 2H, C=OCH_2_); ^13^C-NMR (101 MHz, DMSO-*d*_6_) δ (ppm): 198.40 (*Z*), 191.32 (*E*), 179.28 (*Z*), 178.98 (*E*), 171.16 (*E*), 167.22 (*Z*), 166.75 (*Z*), 165.95 (*E*), 138.21 (*Z*), 137.90 (*E*), 128.53 (2 × C (*E*)), 128.42 (2 × C (*Z*)), 127.99 (*E*), 127.82 (*Z*), 126.91 (2 × C (*Z*)), 126.70 (2 × C (*E*)), 102.09 (*Z*), 101.49 (*E*), 51.15 (*Z*), 50.26 (*E*), 28.45 (*Z*), 27.63 (*E*), 27.52 (*Z*), 27.45 (*E*); EI-MS (*m*/*z*) 256.1 [M]^+^. Anal. Calcd. for C_1__4_H_1__2_N_2_O_3_: C, 65.62; H, 4.72; N, 10.93. Found: C, 65.50; H, 4.59; N, 11.08.

*1-(4-Fluorophenyl)-1′,3,4,5′-tetrahydro-[2,3′-bipyrrolylidene]-2′,4′,5(1H)-trione* (**5b**). White solid (63.4%), m.p. 252.4 °C (decomp). IR (KBr, cm^−1^) ν: 3168, 3056, 2920, 1768, 1712, 1651, 1566, 1454, 1359, 1292, 1141, 947, 768; ^1^H-NMR (400 MHz, DMSO-*d*_6_) δ (ppm): 7.91 (s, (0.8)1H (*E*), NH), 7.43 (s, (0.2)1H (*Z*), NH), 7.29–7.14 (m, 4H, PhH), 3.63 (s, (0.4)2H (*Z*), C*H*_2_NH), 3.54–3.46 (m, (1.6)2H (*E*), C=CCH_2_), 3.44–3.40 (m, (0.4)2H (*Z*), C=CCH_2_), 3.39 (s, (1.6)2H (*E*), C*H*_2_NH), 2.78–2.68 (m, 2H, C=OCH_2_); ^13^C-NMR (101 MHz, DMSO-*d*_6_) δ (ppm): 198.40 (*Z*), 191.62 (*E*), 179.31 (*Z*), 178.99 (*E*), 171.13 (*E*), 167.30 (*Z*), 166.82 (*Z*), 166.10 (*E*), 163.59 (d, *J*_CF_ = 243.5 Hz), 134.55 (d, *J*_CF_ = 3.2 Hz (*Z*)), 134.24 (d, *J*_CF_ = 2.8 Hz (*E*)), 129.12 (d, *J*_CF_ = 9.0 Hz, 2 × C (*Z*)), 128.97 (d, *J*_CF_ = 8.9 Hz, 2 × C (*E*)), 115.34 (d, *J*_CF_ = 23.0 Hz, 2 × C (*E*)), 115.25 (d, *J*_CF_ = 22.9 Hz, 2 × C (*Z*)), 101.95 (*Z*), 101.37 (*E*), 51.12 (*Z*), 50.28 (*E*), 28.40 (*Z*), 27.50 (*E*), 27.41; EI-MS (*m*/*z*) 274.1 [M]^+^. Anal. Calcd. for C_1__4_H_1__1_FN_2_O_3_: C, 61.31; H, 4.04; N, 10.21. Found: C, 61.18; H, 4.18; N, 10.13.

*1-(2-Chlorophenyl)-1′,3,4,5′-tetrahydro-[2,3′-bipyrrolylidene]-2′,4′,5(1H)-trione* (**5c**). White solid (43.9%), m.p. 216.4 °C (decomp). IR (KBr, cm^−1^) ν: 3143, 3057, 2855, 1767, 1716, 1663, 1547, 1450, 1300, 1212, 1149, 966, 716; ^1^H-NMR (400 MHz, DMSO-*d*_6_) δ (ppm): 7.95 (s, (0.8)1H (*E*), NH), 7.53 (d, *J* = 7.9 Hz, 1H, PhH), 7.46 (s, (0.2)1H (*Z*), NH), 7.44–7.27 (m, 3H, PhH), 3.64 (s, (0.4)2H (*Z*), C*H*_2_NH), 3.62–3.45 (m, 2H, C=CCH_2_), 3.39 (s, (1.6)2H (*E*), C*H*_2_NH), 2.86–2.70 (m, 2H, C=OCH_2_); ^13^C-NMR (101 MHz, DMSO-*d*_6_) δ (ppm): 198.39 (*Z*), 192.00 (*E*), 178.53 (*Z*), 178.21 (*E*), 170.79 (*E*), 166.58 (*Z*), 165.70 (*Z*), 164.57 (*E*), 136.05 (*Z*), 135.71 (*E*), 131.96 (*E*), 131.92 (*Z*), 130.37 (*E*), 130.11 (*Z*), 129.54 (*Z*), 129.43 (*E*), 129.34, 127.52 (*E*), 127.43 (*Z*), 102.53 (*Z*), 101.88 (*E*), 51.09 (*Z*), 50.16 (*E*), 28.32 (*Z*), 27.37 (*E*), 27.12 (*E*), 27.00 (*Z*); EI-MS (*m*/*z*) 290.0 [M]^+^. Anal. Calcd. for C_1__4_H_1__1_ClN_2_O_3_: C, 57.84; H, 3.81; N, 9.64. Found: C, 57.99; H, 3.93; N, 9.53.

*1-(3-Chlorophenyl)-1′,3,4,5′-tetrahydro-[2,3′-bipyrrolylidene]-2′,4′,5(1H)-trione* (**5d**). White solid (73.1%), m.p. 244.8 °C (decomp). IR (KBr, cm^−1^) ν: 3368, 3082, 2917, 1758, 1717, 1666, 1560, 1446, 1373, 1299, 1135, 806, 765; ^1^H-NMR (400 MHz, DMSO-*d*_6_) δ (ppm): 7.98 (s, (0.8)1H (*E*), NH), 7.51 (s, (0.2)1H (*Z*), NH), 7.44–7.35 (m, 2H, PhH), 7.31 (d, *J* = 14.8 Hz, 1H, PhH), 7.19 (d, *J* = 3.5 Hz, 1H, PhH), 3.66 (s, (0.4)2H (*Z*), C*H*_2_NH), 3.53–3.47 (m, (1.6)2H (*E*), C=CCH_2_), 3.46–3.42 (m, (0.4)2H (*Z*), C=CCH_2_), 3.41 (s, (1.6)2H (*E*), C*H*_2_NH), 2.78–2.68 (m, 2H, C=OCH_2_); ^13^C-NMR (101 MHz, DMSO-*d*_6_) δ (ppm): 198.38 (*Z*), 191.76 (*E*), 179.08 (*Z*), 178.80 (*E*), 170.98 (*E*), 166.78 (*Z*), 166.64 (*Z*), 165.48 (*E*), 139.51 (*Z*), 139.19 (*E*), 132.62 (*E*), 132.48 (*Z*), 130.09 (*E*), 129.98 (*Z*), 128.10 (*E*), 127.92 (*Z*), 127.13 (*Z*), 127.00 (*E*), 126.04 (*Z*), 125.78 (*E*), 102.11 (*Z*), 101.57 (*E*), 51.13 (*Z*), 50.28 (*E*), 28.38 (*Z*), 27.56 (*E*), 27.45 (*Z*), 27.42 (*E*); EI-MS (*m*/*z*) 290.0 [M]^+^. Anal. Calcd. for C_1__4_H_1__1_ClN_2_O_3_: C, 57.84; H, 3.81; N, 9.64. Found: C, 57.72; H, 3.95; N, 9.55.

*1-(4-Chlorophenyl)-1′,3,4,5′-tetrahydro-[2,3′-bipyrrolylidene]-2′,4′,5(1H)-trione* (**5e**). White solid (61.3%), m.p. 255.1 °C (decomp). IR (KBr, cm^−1^) ν: 3201, 3059, 2920, 1761, 1719, 1666, 1548, 1459, 1296, 1181, 1017, 858, 768; ^1^H-NMR (400 MHz, DMSO-*d*_6_) δ (ppm): 7.94 (s, (0.65)1H (*E*), NH), 7.46 (s, (0.35)1H (*Z*), NH), 7.42 (t, *J* = 8.0 Hz, 2H, PhH), 7.26–7.17 (m, 2H, PhH), 3.64 (s, (0.7)2H (*Z*), C*H*_2_NH), 3.52–3.46 (m, (1.3)2H (*E*), C=CCH_2_), 3.45–3.40 (m, (0.7)2H (*Z*), C=CCH_2_), 3.40 (s, (1.3)2H (*E*), C*H*_2_NH), 2.78–2.68 (m, 2H, C=OCH_2_); ^13^C-NMR (101 MHz, DMSO-*d*_6_) δ (ppm): 198.35 (*Z*), 191.71 (*E*), 179.14 (*Z*), 178.85 (*E*), 171.01 (*E*), 166.95 (*Z*), 166.81 (*Z*), 165.77 (*E*), 137.17 (*Z*), 136.83 (*E*), 132.41 (*E*), 132.27 (*Z*), 128.87 (2 × C (*Z*)), 128.66 (2 × C (*E*)), 128.58 (2 × C (*E*)), 128.49 (2 × C (*Z*)), 102.07 (*Z*), 101.54 (*E*), 51.14 (*Z*), 50.29 (*E*), 28.41 (*Z*), 27.58 (*E*), 27.47 (*Z*), 27.44 (*E*); EI-MS (*m*/*z*) 290.0 [M]^+^. Anal. Calcd. for C_1__4_H_1__1_ClN_2_O_3_: C, 57.84; H, 3.81; N, 9.64. Found: C, 57.68; H, 3.94; N, 9.53.

*1-(4-Bromophenyl)-1′,3,4,5′-tetrahydro-[2,3′-bipyrrolylidene]-2′,4′,5(1H)-trione* (**5f**). White solid (51.6%), m.p. 252.5 °C (decomp). IR (KBr, cm^−1^) ν: 3207, 3061, 2846, 1759, 1713, 1666, 1550, 1488, 1287, 1135, 857, 703; ^1^H-NMR (400 MHz, DMSO-*d*_6_) δ (ppm): 7.95 (s, (0.8)1H (*E*), NH), 7.55 (t, *J* = 7.9 Hz, 2H, PhH), 7.48 (s, (0.2)1H (*Z*), NH), 7.15 (t, *J* = 9.5 Hz, 2H, PhH), 3.64 (s, (0.4)2H (*Z*), C*H*_2_NH), 3.53–3.45 (m, (1.6)2H (*E*), C=CCH_2_), 3.44–3.41 (m, (0.4)2H (*Z*), C=CCH_2_), 3.40 (s, (1.6)2H (*E*), C*H*_2_NH), 2.77–2.68 (m, 2H, C=OCH_2_); ^13^C-NMR (101 MHz, DMSO-*d*_6_) δ (ppm): 198.33 (*Z*), 191.72 (*E*), 179.10 (*Z*), 178.80 (*E*), 171.00 (*E*), 166.88 (*Z*), 166.81 (*Z*), 165.70 (*E*), 137.61 (*Z*), 137.26 (*E*), 131.51 (2 × C (*E*)), 131.43 (2 × C (*Z*)), 129.19 (2 × C (*Z*)), 128.97 (2 × C (*E*)), 120.94 (*E*), 120.80 (*Z*), 102.08 (*Z*), 101.56 (*E*), 51.15 (*Z*), 50.29 (*E*), 28.42 (*Z*), 27.60 (*E*), 27.49 (*Z*), 27.45 (*E*); EI-MS (*m*/*z*) 334.0 [M]^+^. Anal. Calcd. for C_1__4_H_1__1_BrN_2_O_3_: C, 50.17; H, 3.31; N, 8.36. Found: C, 50.07; H, 3.19; N, 8.44.

*1-(o-Tolyl)-1′,3,4,5′-tetrahydro-[2,3′-bipyrrolylidene]-2′,4′,5(1H)-trione* (**5g**). White solid (50.3%), m.p. 233.6 °C (decomp). IR (KBr, cm^−1^) ν: 3149, 3056, 2853, 1758, 1713, 1663, 1545, 1454, 1294, 1147, 964, 857, 779; ^1^H-NMR (400 MHz, DMSO-*d*_6_) δ (ppm): 7.90 (s, (0.6)1H (*E*), NH), 7.35 (s, (0.4)1H (*Z*), NH), 7.28–7.18 (m, 2H, PhH), 7.16–7.09 (m, 1H, PhH), 7.06–6.99 (m, 1H, PhH), 3.62 (s, (0.8)2H (*Z*), C*H*_2_NH), 3.60–3.42 (m, 2H, C=CCH_2_), 3.36 (s, (1.2)2H (*E*), C*H*_2_NH), 2.86–2.65 (m, 2H, 2H, C=OCH_2_), 2.07 (s, (1.8)3H (*Z*), CH_3_), 2.07 (s, (1.2)3H (*E*), CH_3_); ^13^C-NMR (101 MHz, DMSO-*d*_6_) δ (ppm): 198.37 (*Z*), 191.48 (*E*), 179.18 (*Z*), 178.85 (*E*), 171.09 (*E*), 167.31 (*Z*), 166.65 (*Z*), 166.19 (*E*), 137.72 (*Z*), 137.38 (*E*), 136.16 (*Z*), 136.07 (*E*), 130.32 (*E*), 130.21 (*Z*), 128.61 (*E*), 128.39 (*Z*), 126.82 (*Z*), 126.67 (*E*), 126.13 (*E*), 126.06 (*Z*), 102.21 (*Z*), 101.56 (*E*), 51.11 (*Z*), 50.18 (*E*), 28.59 (*Z*), 27.59 (*E*), 27.35 (*E*), 27.24 (*Z*), 18.20 (*E*), 18.19 (*Z*); EI-MS (*m*/*z*) 270.1 [M]^+^. Anal. Calcd. for C_1__5_H_14_N_2_O_3_: C, 66.66; H, 5.22; N, 10.36. Found: C, 66.86; H, 5.33; N, 10.24.

*1-(m-Tolyl)-1′,3,4,5′-tetrahydro-[2,3′-bipyrrolylidene]-2′,4′,5(1H)-trione* (**5h**). White solid (61.6%), m.p. 233.1 °C (decomp). IR (KBr, cm^−1^) ν: 3260, 3057, 2917, 1752, 1718, 1651, 1548, 1441, 1297, 1132, 810, 770; ^1^H-NMR (400 MHz, DMSO-*d*_6_) δ (ppm): 7.91 (s, (0.6)1H (*E*), NH), 7.36 (s, (0.4)1H (*Z*), NH), 7.26–7.19 (m, 1H, PhH), 7.11 (t, *J* = 8.1 Hz, 1H, PhH), 7.02–6.92 (m, 2H, PhH), 3.63 (s, (0.8)2H (*Z*), C*H*_2_NH), 3.51–3.47 (m, (1.2)2H (*E*), C=CCH_2_), 3.44–3.39 (m, (0.8)2H (*Z*), C=CCH_2_), 3.37 (s, (1.2)2H (*E*), C*H*_2_NH), 2.76–2.69 (m, 2H, C=OCH_2_), 2.29 (s, (1.8)3H (*E*), CH_3_), 2.29 (s, (1.2)3H (*Z*), CH_3_); ^13^C-NMR (101 MHz, DMSO-*d*_6_) δ (ppm): 198.42 (*Z*), 191.26 (*E*), 179.32 (*Z*), 178.99 (*E*), 171.19 (*E*), 167.22 (*Z*), 166.72 (*Z*), 165.95 (*E*), 138.11 (*Z*), 137.79 (*E*), 137.68 (*E*), 137.47 (*Z*), 128.68 (*E*), 128.56 (*Z*), 128.31 (*E*), 128.22 (*Z*), 127.33 (*Z*), 127.12 (*E*), 124.10 (*Z*), 123.85 (*E*), 102.08 (*Z*), 101.48 (*E*), 51.14 (*Z*), 50.25 (*E*), 28.44 (*Z*), 27.61 (*E*), 27.49 (*Z*), 27.44 (*E*), 21.39 (*Z*), 21.33 (*E*); EI-MS (*m*/*z*) 270.1 [M]^+^. Anal. Calcd. for C_1__5_H_14_N_2_O_3_: C, 66.66; H, 5.22; N, 10.36. Found: C, 66.48; H, 5.33; N, 10.51.

*1-(p-Tolyl)-1′,3,4,5′-tetrahydro-[2,3′-bipyrrolylidene]-2′,4′,5(1H)-trione* (**5i**). White solid (58.1%), m.p. 273.1 °C (decomp). IR (KBr, cm^−1^) ν: 3206, 3063, 2843, 1749, 1713, 1667, 1553, 1458, 1227, 1136, 963, 705; ^1^H-NMR (400 MHz, DMSO-*d*_6_) δ (ppm): 7.89 (s, (0.65)1H (*E*), NH), 7.35 (s, (0.35)1H (*Z*), NH), 7.14 (t, *J* = 7.9 Hz, 2H, PhH), 7.04 (t, *J* = 8.8 Hz, 2H, PhH), 3.62 (s, (0.7)2H (*Z*), C*H*_2_NH), 3.52–3.46 (m, (1.3)2H (*E*), C=CCH_2_), 3.44–3.39 (m, (0.7)2H (*Z*), C=CCH_2_), 3.37 (s, (1.3)2H (*E*), C*H*_2_NH), 2.77–2.68 (m, 2H, C=OCH_2_), 2.32 (s, 3H, CH_3_); ^13^C-NMR (101 MHz, DMSO-*d*_6_) δ (ppm): 198.35 (*Z*), 191.26 (*E*), 179.35 (*Z*), 179.04 (*E*), 171.19 (*E*), 167.46 (*Z*), 166.75 (*Z*), 166.20 (*E*), 137.14 (*E*), 136.94 (*Z*), 135.68 (*Z*), 135.40 (*E*), 129.03 (2 × C (*E*)), 128.95 (2 × C (*Z*)), 126.64 (2 × C (*Z*)), 126.44 (2 × C (*E*)), 102.02 (*Z*), 101.41 (*E*), 51.15 (*Z*), 50.26 (*E*), 28.43 (*Z*), 27.59 (*E*), 27.48 (*Z*), 27.43 (*E*), 21.27 (*Z*), 21.24 (*E*); EI-MS (*m*/*z*) 270.1 [M]^+^. Anal. Calcd. for C_1__5_H_14_N_2_O_3_: C, 66.66; H, 5.22; N, 10.36. Found: C, 66.78; H, 5.07; N, 10.49.

*1-(3-Methoxyphenyl)-1′,3,4,5′-tetrahydro-[2,3′-bipyrrolylidene]-2′,4′,5(1H)-trione* (**5j**). White solid (64.1%), m.p. 238.4 °C (decomp). IR (KBr, cm^−1^) ν: 3184, 3069, 2838, 1759, 1711, 1662, 1541, 1454, 1241, 1126, 1030, 804, 769; ^1^H-NMR (400 MHz, DMSO-*d*_6_) δ (ppm): 7.92 (s, (0.6)1H (*E*), NH), 7.39 (s, (0.4)1H (*Z*), NH), 7.29–7.20 (m, 1H, PhH), 6.92–6.84 (m, 1H, PhH), 6.79–6.72 (m, 2H, PhH), 3.72 (s, 3H, CH_3_), 3.63 (s, (0.8)2H (*Z*), C*H*_2_NH), 3.52–3.46 (m, (1.2)2H (*E*), C=CCH_2_), 3.44–3.39 (m, (0.8)2H (*Z*), C=CCH_2_), 3.38 (s, (1.2)2H (*E*), C*H*_2_NH), 2.77–2.68 (m, 2H, C=OCH_2_); ^13^C-NMR (101 MHz, DMSO-*d*_6_) δ (ppm): 198.41 (*Z*), 191.39 (*E*), 179.15 (*Z*), 178.86 (*E*), 171.17 (*E*), 167.06 (*Z*), 166.76 (*Z*), 165.75 (*E*), 159.40 (*E*), 159.35 (*Z*), 139.14 (*Z*), 138.86 (*E*), 129.20 (*E*), 129.10 (*Z*), 119.45 (*Z*), 119.27 (*E*), 113.63 (*E*), 113.26 (*Z*), 113.23 (*Z*), 112.82 (*E*), 102.15 (*Z*), 101.50 (*E*), 55.67 (*E*), 55.63 (*Z*), 51.15 (*Z*), 50.25 (*E*), 28.41 (*Z*), 27.59 (*E*), 27.50 (*Z*), 27.40 (*E*); EI-MS (*m*/*z*) 286.1 [M]^+^. Anal. Calcd. for C_1__5_H_14_N_2_O_4_: C, 62.93; H, 4.93; N, 9.79. Found: C, 62.82; H, 4.79; N, 9.64.

*1-(3-(Trifluoromethyl)phenyl)-1′,3,4,5′-tetrahydro-[2,3′-bipyrrolylidene]-2′,4′,5(1H)-trione* (**5k**). White solid (30.3%), m.p. 225.8 °C (decomp). IR (KBr, cm^−1^) ν: 3163, 3058, 2879, 1759, 1718, 1667, 1545, 1455, 1326, 1143, 1108, 859, 791; ^1^H-NMR (400 MHz, DMSO-*d*_6_) δ (ppm): 7.97 (s, (0.7)1H (*E*), NH), 7.69 (t, *J* = 10.2 Hz, 1H, PhH), 7.61 (t, *J* = 11.4 Hz, 2H, PhH), 7.51 (d, *J* = 7.8 Hz, 1H, PhH), 7.48 (s, (0.3)1H (*Z*), NH), 3.65 (s, (0.6)2H (*Z*), C*H*_2_NH), 3.54–3.50 (m, (1.4)2H (*E*), C=CCH_2_), 3.47–3.42 (m, (0.6)2H (*Z*), C=CCH_2_), 3.38 (s, (1.4)2H (*E*), C*H*_2_NH), 2.79–2.70 (m, 2H, C=OCH_2_); ^13^C-NMR (101 MHz, DMSO-*d*_6_) δ (ppm): 198.42 (*Z*), 191.87 (*E*), 179.23 (*Z*), 178.91 (*E*), 170.94 (*E*), 166.82 (*Z*), 166.70 (*Z*), 165.61 (*E*), 138.96 (*Z*), 138.62 (*E*), 131.34 (*Z*), 131.04 (*E*), 129.75 (*E*), 129.59 (*Z*), 129.28 (q, *J*_CF_ = 32.0 Hz (*E*)), 129.16 (q, *J*_CF_ = 32.0 Hz (*Z*)), 124.82 (q, *J*_CF_ = 3.9 Hz (*E*)), 124.66 (q, *J*_CF_ = 3.7 Hz (*Z*)), 124.46 (q, *J*_CF_ = 272.3 Hz), 124.20 (q, *J*_CF_ = 4.0 Hz (*Z*)), 124.02 (q, *J*_CF_ = 4.1 Hz (*E*)), 102.00 (*Z*), 101.49 (*E*), 51.13 (*Z*), 50.24 (*E*), 28.83 (*Z*), 28.42 (*E*), 27.58 (*Z*), 27.47 (*E*); EI-MS (*m*/*z*) 324.1 [M]^+^. Anal. Calcd. for C_1__5_H_1__1_F_3_N_2_O_3_: C, 55.56; H, 3.42; N, 8.64. Found: C, 55.47; H, 3.52; N, 8.54.

### 3.5. General Procedure for the Synthesis of Compounds ***6***

The chloroformate (3.2 mmol) was added dropwise to a mixture of compound **5** (1.5 mmol) in Et_3_N (0.34 g, 3.4 mmol) and chloroform (25 mL) at 0 °C. The resulting mixture was stirred at 0 °C for 0.5–2 h. Then the mixture was washed with water, dried with Na2SO4, filtered and concentrated *in vacuo*. The desired products **6** were obtained by purification on a silica gel column with petroleum ether/ethyl acetate (*v*/*v*, 3:1).

*1-(3-Chlorophenyl)-2′-oxo-2′,5′-dihydro-1H,1′H-[2,3′-bipyrrole]-4′,5-diyl dimethyl bis(carbonate)* (**6a**). White solid (50.1%), m.p. 164.6–166.2 °C. IR (KBr, cm^−1^) ν: 3198, 3073, 2962, 2863, 1762, 1690, 1557, 1431, 1375, 1235, 928, 773; ^1^H-NMR (400 MHz, CDCl_3_) δ (ppm): 7.35–7.28 (m, 3H, PhH), 7.13–7.07 (m, 1H, PhH), 6.57 (d, *J* = 4.0 Hz, 1H, ArH), 6.39–6.22 (brs, 1H, NH), 6.10 (d, *J* = 4.0 Hz, 1H, ArH), 4.24 (s, 2H, C*H*_2_NH), 3.79 (s, 3H, CH_3_), 3.76 (s, 3H, CH_3_); ^13^C-NMR (101 MHz, CDCl_3_) δ (ppm): 171.40, 156.62, 152.73, 150.93, 138.32, 137.57, 134.31, 129.87, 127.67, 127.23, 125.22, 116.34, 111.49 (2 × C), 96.98, 55.94, 55.89, 45.38; EI-MS (*m*/*z*) 406.1 [M]^+^. Anal. Calcd. for C_1__8_H_1__5_ClN_2_O_7_: C, 53.15; H, 3.72; N, 6.89. Found: C, 53.02; H, 3.80; N, 6.81.

*1-(3-Chlorophenyl)-2′-oxo-2′,5′-dihydro-1H,1′H-[2,3′-bipyrrole]-4′,5-diyl diethyl bis(carbonate)* (**6b**). White solid (82.4%), m.p. 138.1–140.0 °C. IR (KBr, cm^−1^) ν: 3188, 3081, 2993, 2863, 1764, 1693, 1592, 1490, 1367, 1211, 881, 770; ^1^H-NMR (400 MHz, CDCl_3_) δ (ppm): 7.34 (s, 1H, PhH), 7.28 (dd, *J* = 3.9, 1.7 Hz, 2H, PhH), 7.14–7.08 (m, 1H, PhH), 6.75–6.35 (brs, 1H, NH), 6.55 (d, *J* = 4.0 Hz, 1H, ArH), 6.08 (d, *J* = 4.0 Hz, 1H, ArH), 4.24 (s, 2H, NHC*H*_2_), 4.18 (q, *J* = 7.1 Hz, 2H, OCH_2_), 4.14 (q, *J* = 7.1 Hz, 2H, OCH_2_), 1.32 (t, *J* = 7.1 Hz, 3H, CH_3_), 1.24 (t, *J* = 7.1 Hz, 3H, CH_3_); ^13^C-NMR (101 MHz, CDCl_3_) δ (ppm): 171.51, 156.87, 152.19, 150.27, 138.28, 137.69, 134.22, 129.84, 127.60, 127.07, 125.19, 116.42, 111.38, 111.30, 97.13, 65.74, 65.48, 45.46, 14.00, 13.94; EI-MS (*m*/*z*) 434.1 [M]^+^. Anal. Calcd. for C_20_H_1__9_ClN_2_O_7_: C, 55.24; H, 4.40; N, 6.44. Found: C, 55.34; H, 4.29; N, 6.33.

*1-(3-Chlorophenyl)-2′-oxo-2′,5′-dihydro-1H,1′H-[2,3′-bipyrrole]-4′,5-diyl dipropyl bis(carbonate)* (**6c**). White solid (69.3%), m.p. 102.0–103.0 °C. IR (KBr, cm^−1^) ν: 3221, 3076, 2970, 2877, 1765, 1695, 1567, 1477, 1204, 1022, 927, 771; ^1^H-NMR (400 MHz, CDCl_3_) δ (ppm): 7.34–7.31 (m, 1H, PhH), 7.28 (dd, *J* = 3.9, 1.9 Hz, 2H, PhH), 7.14–7.10 (m, 1H, PhH), 6.54 (d, *J* = 4.0 Hz, 1H, ArH), 6.42–6.19 (brs, 1H, NH), 6.07 (d, *J* = 4.0 Hz, 1H, ArH), 4.24 (s, 2H, NHC*H*_2_), 4.09 (t, *J* = 6.7 Hz, 2H, OCH_2_), 4.04 (t, *J* = 6.8 Hz, 2H, OCH_2_), 1.75–1.66 (m, 2H, C*H*_2_CH_3_), 1.66–1.57 (m, 2H, C*H*_2_CH_3_), 0.96 (t, *J* = 7.4 Hz, 3H, CH_3_), 0.89 (t, *J* = 7.4 Hz, 3H, CH_3_); ^13^C-NMR (101 MHz, CDCl_3_) δ (ppm): 171.51, 157.03, 152.37, 150.41, 138.29, 137.68, 134.22, 129.81, 127.61, 127.04, 125.19, 116.40, 111.36 (2 × C), 97.18, 71.21, 70.97, 45.48, 21.79, 21.72, 10.10, 9.97; EI-MS (*m*/*z*) 462.1 [M]^+^. Anal. Calcd. for C_22_H_23_ClN_2_O_7_: C, 57.09; H, 5.01; N, 6.05. Found: C, 57.21; H, 5.11; N, 6.23.

*1-(3-Chlorophenyl)-2′-oxo-2′,5′-dihydro-1H,1′H-[2,3′-bipyrrole]-4′,5-diyl diisopropyl bis(carbonate)* (**6d**). White solid (40.2%), m.p. 122.1–124.1 °C. IR (KBr, cm^−1^) ν: 3187, 3059, 2997, 2877, 1768, 1689, 1593, 1420, 1339, 1226, 1025, 904, 752; ^1^H-NMR (400 MHz, CDCl_3_) δ (ppm): 7.33 (s, 1H, PhH), 7.29–7.25 (m, 2H, PhH), 7.15–7.09 (m, 1H, PhH), 6.52 (d, *J* = 3.9 Hz, 1H, ArH), 6.39–6.11 (brs, 1H, NH), 6.06 (d, *J* = 4.0 Hz, 1H, ArH), 4.87–4.71 (m, 2H, 2 × OCH), 4.26 (s, 2H, CH_2_), 1.30 (d, *J* = 6.3 Hz, 6H, 2 × CH_3_), 1.21 (d, *J* = 6.3 Hz, 6H, 2 × CH_3_); ^13^C-NMR (101 MHz, CDCl_3_) δ (ppm): 171.47, 157.32, 151.79, 149.75, 138.23, 137.84, 134.16, 129.75, 127.56, 126.89, 125.08, 116.47, 111.22, 110.95, 97.28, 74.41, 73.90, 45.57, 21.46 (2 × C), 21.40 (2 × C); EI-MS (*m*/*z*) 462.1 [M]^+^. Anal. Calcd. for C_22_H_23_ClN_2_O_7_: C, 57.09; H, 5.01; N, 6.05. Found: C, 57.17; H, 5.15; N, 6.16.

*Dibutyl (1-(3-chlorophenyl)-2′-oxo-2′,5′-dihydro-1H,1′H-[2,3′-bipyrrole]-4′,5-diyl) bis(carbonate)* (**6e**). White solid (51.3%), m.p. 81.7–83.5 °C. IR (KBr, cm^−1^) ν: 3199, 3079, 2962, 2874, 1765, 1697, 1594, 1492, 1204, 1017, 906, 772; ^1^H-NMR (400 MHz, CDCl_3_) δ (ppm): 7.34–7.31 (m, 1H, PhH), 7.30–7.27 (m, 2H, PhH), 7.13–7.09 (m, 1H, PhH), 6.68–6.55 (brs, 1H, NH), 6.54 (d, *J* = 4.0 Hz, 1H, ArH), 6.07 (d, *J* = 4.0 Hz, 1H, ArH), 4.24 (s, 2H, NHC*H*_2_), 4.13 (t, *J* = 6.6 Hz, 2H, OCH_2_), 4.08 (t, *J* = 6.7 Hz, 2H, OCH_2_), 1.70–1.61 (m, 2H, OCH_2_C*H*_2_), 1.61–1.52 (m, 2H, OCH_2_C*H_2_*), 1.45–1.35 (m, 2H, C*H_2_*CH_3_), 1.34–1.25 (m, 2H, C*H*_2_CH_3_), 0.95 (t, *J* = 7.4 Hz, 3H, CH_2_C*H*_3_), 0.90 (t, *J* = 7.4 Hz, 3H, CH_2_C*H*_3_); ^13^C-NMR (101 MHz, CDCl_3_) δ (ppm): 171.43, 157.00, 152.37, 150.42, 138.29, 137.68, 134.22, 129.79, 127.59, 127.04, 125.20, 116.40, 111.37, 111.33, 97.19, 69.59, 69.30, 45.46, 30.40, 30.30, 18.83, 18.69, 13.63, 13.57; EI-MS (*m*/*z*) 490.2 [M]^+^. Anal. Calcd. for C_24_H_27_ClN_2_O_7_: C, 58.72; H, 5.54; N, 5.71. Found: C, 58.58; H, 5.71; N, 5.62.

*1-(3-Chlorophenyl)-2′-oxo-2′,5′-dihydro-1H,1′H-[2,3′-bipyrrole]-4′,5-diyl diisobutyl bis(carbonate)* (**6f**). White solid (75.3%), m.p. 104.8–106.0 °C. IR (KBr, cm^−1^) ν: 3188, 3073, 2966, 2876, 1769, 1689, 1560, 1473, 1368, 1206, 1032, 933, 747; ^1^H-NMR (400 MHz, CDCl_3_) δ (ppm): 7.33–7.31 (m, 1H, PhH), 7.28 (dd, *J* = 3.9, 1.9 Hz, 2H, PhH), 7.15–7.09 (m, 1H, PhH), 6.54 (d, *J* = 4.0 Hz, 1H, ArH), 6.42–6.17 (brs, 1H, NH), 6.07 (d, *J* = 4.0 Hz, 1H, ArH), 4.24 (s, 2H, NHC*H*_2_), 3.90 (d, *J* = 6.7 Hz, 2H, OCH_2_), 3.86 (d, *J* = 6.7 Hz, 2H, OCH_2_), 2.06–1.84 (m, 2H, 2 × C*H*CH_3_), 0.95 (d, *J* = 6.7 Hz, 6H, 2 × CH_3_), 0.87 (d, *J* = 6.7 Hz, 6H, 2 × CH_3_); ^13^C-NMR (101 MHz, CDCl_3_) δ (ppm): 171.38, 157.10, 152.45, 150.48, 138.29, 137.67, 134.22, 129.80, 127.61, 127.00, 125.19, 116.38, 111.41, 111.38, 97.24, 75.58, 75.33, 45.43, 27.66, 27.60, 18.82 (2 × C), 18.66 (2 × C); EI-MS (*m*/*z*) 490.2 [M]^+^. Anal. Calcd. for C_24_H_27_ClN_2_O_7_: C, 58.72; H, 5.54; N, 5.71. Found: C, 58.59; H, 5.39; N, 5.85.

*Dibenzyl (1-(3-chlorophenyl)-2′-oxo-2′,5′-dihydro-1H,1′H-[2,3′-bipyrrole]-4′,5-diyl) bis(carbonate)* (**6g**). White solid (30.7%), m.p. 132.2–135.0 °C. IR (KBr, cm^−1^) ν: 3186, 3074, 2877, 1767, 1688, 1560, 1496, 1318, 1204, 1033, 903, 772; ^1^H-NMR (400 MHz, CDCl_3_) δ (ppm): 7.42–7.31 (m, 9H, PhH), 7.25 (d, *J* = 6.4 Hz, 2H, PhH), 7.18 (d, *J* = 7.9 Hz, 1H, PhH), 7.11 (t, *J* = 7.9 Hz, 1H, PhH), 7.05 (d, *J* = 8.0 Hz, 1H, PhH), 6.52 (d, *J* = 4.0 Hz, 1H, ArH), 6.25–6.10 (brs, 1H, NH), 6.07 (d, *J* = 4.0 Hz, 1H, ArH), 5.12 (s, 2H, OCH_2_), 5.09 (s, 2H, OCH_2_), 4.22 (s, 2H, NHC*H*_2_); ^13^C-NMR (101 MHz, CDCl_3_) δ (ppm): 171.19, 156.88, 152.25, 150.27, 138.23, 137.50, 134.30, 134.21, 133.83, 129.80, 129.11, 128.81, 128.78 (2 × C), 128.67 (4 × C), 128.41 (2 × C), 127.69, 127.03, 125.15, 116.33, 111.46 (2 × C), 97.20, 71.12, 70.83, 45.43; EI-MS (*m*/*z*) 502.0 [M − 56]^+^. Anal. Calcd. for C_30_H_23_ClN_2_O_7_: C, 64.46; H, 4.15; N, 5.01. Found: C, 64.36; H, 4.04; N, 5.14.

*Diisobutyl (2′-oxo-1-phenyl-2′,5′-dihydro-1H,1′H-[2,3′-bipyrrole]-4′,5-diyl) bis(carbonate)* (**6h**). White solid (32.6%), m.p. 95.0–96.8 °C. IR (KBr, cm^−1^) ν: 3198, 3085, 2963, 2874, 1766, 1695, 1560, 1498, 1367, 1204, 1029, 922, 774; ^1^H-NMR (400 MHz, CDCl_3_) δ (ppm): 7.38–7.32 (m, 2H, PhH), 7.30 (d, *J* = 7.0 Hz, 1H, PhH), 7.25 (d, *J* = 8.7 Hz, 2H, PhH), 6.53 (d, *J* = 4.0 Hz, 1H, ArH), 6.35–6.10 (brs, 1H, NH), 6.07 (d, *J* = 4.0 Hz, 1H, ArH), 4.21 (s, 2H, NHC*H_2_*), 3.86 (d, *J* = 6.7 Hz, 2H, OCH_2_), 3.81 (d, *J* = 6.6 Hz, 2H, OCH_2_), 2.01–1.82 (m, 2H, 2 × C*H*CH_3_), 0.96 (d, *J* = 6.7 Hz, 6H, 2 × CH_3_), 0.84 (d, *J* = 6.7 Hz, 6H, 2 × CH_3_); ^13^C-NMR (101 MHz, CDCl_3_) δ (ppm): 171.74, 156.97, 152.53, 150.46, 138.34, 136.49, 128.77 (2 × C), 127.35, 126.86 (2 × C), 116.32, 111.73, 110.91, 96.91, 75.30, 75.15, 45.43, 27.61, 27.58, 18.84 (2 × C), 18.67 (2 × C); EI-MS (*m/z*) 456.2 [M]^+^. Anal. Calcd. for C_24_H_28_N_2_O_7_: C, 63.15; H, 6.18; N, 6.14. Found: C, 63.04; H, 6.07; N, 6.26.

*1-(4-Fluorophenyl)-2′-oxo-2′,5′-dihydro-1H,1′H-[2,3′-bipyrrole]-4′,5-diyl diisobutyl bis(carbonate)* (**6i**). White solid (48.6%), m.p. 93.9–95.1 °C. IR (KBr, cm^−1^) ν: 3208, 3079, 2964, 2874, 1767, 1690, 1509, 1466, 1370, 1205, 1099, 842, 775; ^1^H-NMR (400 MHz, CDCl_3_) δ (ppm): 7.25 (dd, *J* = 8.5, 4.9 Hz, 2H, PhH), 7.03 (t, *J* = 8.4 Hz, 2H, PhH), 6.50 (d, *J* = 3.7 Hz, 1H, ArH), 6.27–6.19 (brs, 1H, NH), 6.06 (d, *J* = 3.8 Hz, 1H, ArH), 4.22 (s, 2H, NHC*H_2_*), 3.87 (t, *J* = 6.9 Hz, 4H, 2 × OCH_2_), 2.02–1.82 (m, 2H, 2 × C*H*CH_3_), 0.96 (d, *J* = 6.7 Hz, 6H, 6H, 2 × CH_3_), 0.86 (d, *J* = 6.7 Hz, 6H, 6H, 2 × CH_3_); ^13^C-NMR (101 MHz, CDCl_3_) δ (ppm): 171.48, 161.69 (d, *J*_C-F_ = 247.3 Hz), 157.32, 152.50, 150.52, 138.38, 132.50 (d, *J*_C-F_ = 2.9 Hz), 128.76 (d, *J*_C-F_ = 8.6 Hz, 2 × C), 116.40, 115.66 (d, *J*_C-F_ = 22.8 Hz, 2 × C), 111.65, 110.96, 96.91, 75.47, 75.24, 45.48, 27.63, 27.62, 18.81 (2 × C), 18.64 (2 × C); EI-MS (*m*/*z*) 474.2 [M]^+^. Anal. Calcd. for C_24_H_27_FN_2_O_7_: C, 60.75; H, 5.74; N, 5.90. Found: C, 60.83; H, 5.60; N, 5.78.

*1-(2-Chlorophenyl)-2′-oxo-2′,5′-dihydro-1H,1′H-[2,3′-bipyrrole]-4′,5-diyl diisobutyl bis(carbonate)* (**6j**). White solid (46.2%), m.p. 107.2–109.1 °C. IR (KBr, cm^−1^) ν: 3204, 3082, 2966, 2872, 1769, 1694, 1559, 1491, 1370, 1208, 990, 765; ^1^H-NMR (400 MHz, CDCl_3_) δ (ppm): 7.44 (d, *J* = 8.0 Hz, 1H, PhH), 7.34–7.21 (m, 3H, PhH), 6.57 (d, *J* = 4.0 Hz, 1H, ArH), 6.23–6.14 (brs, 1H, NH), 6.12 (d, *J* = 4.0 Hz, 1H, ArH), 4.21 (s, 2H, C*H_2_*NH), 3.90 (dd, *J* = 6.8, 1.2 Hz, 2H, OCH_2_), 3.86 (dd, *J* = 6.6, 2.7 Hz, 2H, OCH_2_), 2.03–1.84 (m, 2H, 2 × C*H*CH_3_), 0.98 (d, *J* = 6.7 Hz, 6H, 2 × CH_3_), 0.85 (d, *J* = 6.7 Hz, 6H, 2 × CH_3_); ^13^C-NMR (101 MHz, CDCl_3_) δ (ppm): 171.47, 157.21, 152.19, 150.60, 138.46, 134.22, 133.25, 130.33, 130.08, 129.32, 126.95, 116.48, 111.29, 111.10, 96.82, 75.44, 75.14, 45.36, 27.64, 27.61, 18.86 (2 × C), 18.67 (2 × C); EI-MS (*m*/*z*) 490.2 [M]^+^. Anal. Calcd. for C_24_H_27_ClN_2_O_7_: C, 58.72; H, 5.54; N, 5.71. Found: C, 58.85; H, 5.66; N, 5.81.

*1-(4-Chlorophenyl)-2′-oxo-2′,5′-dihydro-1H,1′H-[2,3′-bipyrrole]-4′,5-diyl diisobutyl bis(carbonate)* (**6k**). White solid (59.2%), m.p. 123.7–125.1 °C. IR (KBr, cm^−1^) ν: 3223, 3079, 2964, 2874, 1767, 1685, 1560, 1494, 1204, 1091, 831, 776; ^1^H-NMR (400 MHz, CDCl_3_) δ (ppm): 7.32 (d, *J* = 8.7 Hz, 2H, PhH), 7.21 (d, *J* = 8.7 Hz, 2H, PhH), 6.53 (d, *J* = 4.0 Hz, 1H, ArH), 6.42–6.23 (brs, 1H, NH), 6.07 (d, *J* = 4.0 Hz, 1H, ArH), 4.24 (s, 2H, NHC*H_2_*), 3.89 (d, *J* = 6.7 Hz, 2H, OCH_2_), 3.86 (d, *J* = 6.6 Hz, 2H, OCH_2_), 2.02–1.83 (m, 2H, 2 × C*H*CH_3_), 0.96 (d, *J* = 6.7 Hz, 6H, 2 × CH_3_), 0.86 (d, *J* = 6.7 Hz, 6H, 2 × CH_3_); ^13^C-NMR (101 MHz, CDCl_3_) δ (ppm): 171.42, 157.05, 152.42, 150.50, 138.28, 135.14, 133.22, 129.01 (2 × C), 128.14 (2 × C), 116.35, 111.55, 111.28, 97.08, 75.50, 75.28, 45.45, 27.64, 27.61, 18.80 (2 × C), 18.63 (2 × C); EI-MS (*m*/*z*) 490.2 [M]^+^. Anal. Calcd. for C_24_H_27_ClN_2_O_7_: C, 58.72; H, 5.54; N, 5.71. Found: C, 58.56; H, 5.39; N, 5.79.

*1-(4-bromophenyl)-2′-oxo-2′,5′-dihydro-1H,1′H-[2,3′-bipyrrole]-4′,5-diyl diisobutyl bis(carbonate)* (**6l**). White solid (60.0%), m.p. 126.7–128.7 °C. IR (KBr, cm^−1^) ν: 3233, 3073, 2964, 2869, 1768, 1685, 1560, 1491, 1371, 1211, 994, 775; ^1^H-NMR (400 MHz, CDCl_3_) δ (ppm): 7.47 (d, *J* = 8.7 Hz, 2H, PhH), 7.15 (d, *J* = 8.7 Hz, 2H, PhH), 6.60–6.44 (brs, 1H, NH), 6.54 (d, *J* = 4.0 Hz, 1H, ArH), 6.07 (d, *J* = 4.0 Hz, 1H, ArH), 4.24 (s, 2H, NHC*H_2_*), 3.89 (d, *J* = 6.7 Hz, 2H, OCH_2_), 3.86 (d, *J* = 6.6 Hz, 2H, OCH_2_), 2.01–1.92 (m, 1H, C*H*CH_3_), 1.91–1.83 (m, 1H, C*H*CH_3_), 0.97 (d, *J* = 6.7 Hz, 6H, 2 × CH_3_), 0.87 (d, *J* = 6.7 Hz, 6H, 2 × CH_3_); ^13^C-NMR (101 MHz, CDCl_3_) δ (ppm): 171.45, 156.94, 152.41, 150.49, 138.21, 135.65, 132.02 (2 × C), 128.42 (2 × C), 121.19, 116.31, 111.47, 111.35, 97.14, 75.53, 75.29, 45.47, 27.64, 27.62, 18.83 (2 × C), 18.64 (2 × C); EI-MS (*m*/*z*) 534.1 [M]^+^. Anal. Calcd. for C_24_H_27_BrN_2_O_7_: C, 53.84; H, 5.08; N, 5.23. Found: C, 53.71; H, 4.97; N, 5.12.

*Diisobutyl (2′-oxo-1-(o-tolyl)-2′,5′-dihydro-1H,1′H-[2,3′-bipyrrole]-4′,5-diyl) bis(carbonate)* (**6m**). White solid (66.6%), m.p. 91.3–92.9 °C. IR (KBr, cm^−1^) ν: 3204, 3085, 2965, 2874, 1770, 1693, 1553, 1496, 1376, 1208, 989, 770; ^1^H-NMR (400 MHz, CDCl_3_) δ (ppm): 7.25–7.10 (m, 4H, PhH), 6.70–6.54 (brs, 1H, NH), 6.52 (d, *J* = 4.0 Hz, 1H, ArH), 6.08 (d, *J* = 4.0 Hz, 1H, ArH), 4.18 (s, 2H, NHC*H*_2_), 3.87–3.80 (m, 4H, 2 × OCH_2_), 2.10 (s, 3H, PhCH_3_), 2.04–1.92 (m, 1H, C*H*CH_3_), 1.88–1.76 (m, 1H, C*H*CH_3_), 0.98 (d, *J* = 6.7 Hz, 6H, 2 × CHC*H*_3_), 0.80 (d, *J* = 6.7 Hz, 6H, 2 × CHC*H*_3_); ^13^C-NMR (101 MHz, CDCl_3_) δ (ppm): 171.74, 157.27, 152.45, 150.54, 138.36, 136.90, 135.24, 130.56, 128.89, 128.28, 125.92, 116.48, 111.53, 110.47, 96.57, 75.35, 75.00, 45.35, 27.63, 27.57, 18.88, 18.87, 18.62 (2 × C), 17.55; EI-MS (*m*/*z*) 470.2 [M]^+^. Anal. Calcd. for C_25_H_30_N_2_O_7_: C, 63.82; H, 6.43; N, 5.95. Found: C, 63.65; H, 6.31; N, 5.83.

*Diisobutyl (2′-oxo-1-(m-tolyl)-2′,5′-dihydro-1H,1′H-[2,3′-bipyrrole]-4′,5-diyl) bis(carbonate)* (**6n**). White solid (59.7%), m.p. 82.4–84.0 °C. IR (KBr, cm^−1^) ν: 3185, 3079, 2967, 2874, 1767, 1689, 1560, 1492, 1369, 1208, 1033, 933, 744; ^1^H-NMR (400 MHz, CDCl_3_) δ (ppm): 7.21 (dd, *J* = 10.9, 5.3 Hz, 1H, PhH), 7.07 (dd, *J* = 15.2, 6.8 Hz, 3H, PhH), 6.52 (d, *J* = 4.0 Hz, 1H, ArH), 6.46–6.27 (brs, 1H, NH), 6.05 (d, *J* = 4.0 Hz, 1H, ArH), 4.21 (s, 2H, NHC*H*_2_), 3.88 (d, *J* = 6.7 Hz, 2H, OCH_2_), 3.81 (d, *J* = 6.7 Hz, 2H, OCH_2_), 2.32 (s, 3H, PhCH_3_), 2.00–1.81 (m, 2H, 2 × C*H*CH_3_), 0.95 (d, *J* = 6.7 Hz, 6H, 2 × CHC*H*_3_), 0.85 (d, *J* = 6.7 Hz, 6H, 2 × CHC*H*_3_); ^13^C-NMR (101 MHz, CDCl_3_) δ (ppm): 171.76, 156.83, 152.58, 150.51, 138.66, 138.33, 136.37, 128.51, 128.08, 127.25, 123.83, 116.30, 111.86, 110.87, 96.86, 75.32, 75.12, 45.37, 27.64, 27.60, 21.29, 18.83 (2 × C), 18.66 (2 × C); EI-MS (*m*/*z*) 470.2 [M]^+^. Anal. Calcd. for C_25_H_30_N_2_O_7_: C, 63.82; H, 6.43; N, 5.95. Found: C, 64.02; H, 6.55; N, 6.10.

*Diisobutyl (2′-oxo-1-(p-tolyl)-2′,5′-dihydro-1H,1′H-[2,3′-bipyrrole]-4′,5-diyl) bis(carbonate)* (**6o**). White solid (40.2%), m.p. 94.7–96.6 °C. IR (KBr, cm^−1^) ν: 3204, 3082, 2964, 2874, 1766, 1689, 1516, 1469, 1369, 1212, 1107, 995, 774; ^1^H-NMR (400 MHz, CDCl_3_) δ (ppm): 7.12 (s, 4H, PhH), 6.72–6.55 (brs, 1H, NH), 6.49 (d, *J* = 4.0 Hz, 1H, ArH), 6.04 (d, *J* = 4.0 Hz, 1H, ArH), 4.21 (s, 2H, NHC*H*_2_), 3.87 (d, *J* = 6.7 Hz, 2H, OCH_2_), 3.81 (d, *J* = 6.6 Hz, 2H, OCH_2_), 2.34 (s, 3H, PhCH_3_), 2.01–1.81 (m, 2H, 2 × C*H*CH_3_), 0.96 (d, *J* = 6.7 Hz, 6H, 2 × CHC*H*_3_), 0.85 (d, *J* = 6.7 Hz, 6H, 2 × CHC*H*_3_); ^13^C-NMR (101 MHz, CDCl_3_) δ (ppm): 171.79, 157.01, 152.57, 150.52, 138.38, 137.10, 133.89, 129.36 (2 × C), 126.63 (2 × C), 116.31, 111.89, 110.68, 96.72, 75.26, 75.10, 45.42, 27.62, 27.60, 21.12, 18.82 (2 × C), 18.65 (2 × C); EI-MS (*m*/*z*) 470.2 [M]^+^. Anal. Calcd. for C_25_H_30_N_2_O_7_: C, 63.82; H, 6.43; N, 5.95. Found: C, 64.00; H, 6.32; N, 6.11.

*Diisobutyl (1-(3-methoxyphenyl)-2′-oxo-2′,5′-dihydro-1H,1′H-[2,3′-bipyrrole]-4′,5-diyl) bis(carbonate)* (**6p**). White solid (61.2%), m.p. 102.5–103.8 °C. IR (KBr, cm^−1^) ν: 3204, 3068, 2964, 2872, 1766, 1686, 1560, 1494, 1369, 1206, 1030, 937, 784; ^1^H-NMR (400 MHz, CDCl_3_) δ (ppm): 7.23 (t, *J* = 8.0 Hz, 1H, PhH), 6.83 (d, *J* = 8.0 Hz, 3H, PhH), 6.53 (d, *J* = 4.0 Hz, 1H, ArH), 6.45–6.32 (brs, 1H, NH), 6.06 (d, *J* = 4.0 Hz, 1H, ArH), 4.22 (s, 2H, NHC*H*_2_), 3.88 (d, *J* = 6.7 Hz, 2H, OCH_2_), 3.81 (d, *J* = 6.7 Hz, 2H, OCH_2_), 3.76 (s, 3H, PhOCH_3_), 1.99–1.82 (m, 2H, 2 × C*H*CH_3_), 0.94 (d, *J* = 6.7 Hz, 6H, 2 × CHC*H*_3_), 0.86 (d, *J* = 6.7 Hz, 6H, 2 × CHC*H*_3_); ^13^C-NMR (101 MHz, CDCl_3_) δ (ppm): 171.69, 159.77, 156.95, 152.57, 150.54, 138.31, 137.52, 129.42, 119.00, 116.34, 113.57, 112.09, 111.90, 111.00, 96.97, 75.38, 75.17, 55.35, 45.39, 27.64, 27.56, 18.81 (2 × C), 18.67 (2 × C); EI-MS (*m*/*z*) 486.2 [M]^+^. Anal. Calcd. for C_25_H_30_N_2_O_8_: C, 61.72; H, 6.22; N, 5.76. Found: C, 61.53; H, 6.32; N, 5.91.

*Diisobutyl (2′-oxo-1-(3-(trifluoromethyl)phenyl)-2′,5′-dihydro-1H,1′H-[2,3′-bipyrrole]-4′,5-diyl) bis(carbonate)* (**6q**). White solid (76.7%), m.p. 81.4–83.5 °C. IR (KBr, cm^−1^) ν: 3190, 3078, 2970, 2878, 1770, 1690, 1546, 1496, 1335, 1209, 1125, 932, 750; ^1^H-NMR (400 MHz, CDCl_3_) δ (ppm): 7.61–7.54 (m, 2H, PhH), 7.47 (d, *J* = 7.7 Hz, 1H, PhH), 7.44 (s, 1H, PhH), 6.54 (d, *J* = 4.0 Hz, 1H, ArH), 6.47–6.32 (brs, 1H, NH), 6.10 (d, *J* = 4.0 Hz, 1H, ArH), 4.23 (s, 2H, NHC*H*_2_), 3.89 (d, *J* = 6.7 Hz, 2H, OCH_2_), 3.84 (d, *J* = 6.8 Hz, 2H, OCH_2_), 2.00–1.81 (m, 2H, 2 × C*H*CH_3_), 0.93 (d, *J* = 6.7 Hz, 6H, 2 × CHC*H*_3_), 0.85 (d, *J* = 6.7 Hz, 6H, 2 × CHC*H*_3_); ^13^C-NMR (101 MHz, CDCl_3_) δ (ppm): ^13^C-NMR (101 MHz, CDCl3) δ (ppm): 171.24, 157.53, 152.38, 150.45, 138.24, 137.16, 131.31 (q, *J*_CF_ = 32.9 Hz), 130.16, 129.47, 124.06 (q, *J*_CF_ = 3.7 Hz), 123.72 (q, *J*_CF_ = 3.9 Hz), 123.59 (q, *J*_CF_ = 272.5 Hz), 116.27, 111.51, 111.38, 97.32, 75.56, 75.36, 45.46, 27.62, 27.52, 18.72 (2 × C), 18.58 (2 × C); EI-MS (*m*/*z*) 524.2 [M]^+^. Anal. Calcd. for C_25_H_27_F_3_N_2_O_7_: C, 57.25; H, 5.19; N, 5.34. Found: C, 57.11; H, 5.31; N, 5.18.

### 3.6. Antifungal Activity Test

Compounds **4**–**6** were screened *in vitro* for antifungal activity against the phytopathogenic fungi *F.* *graminearum*, *B.* *cinerea*, and *R.* *solani* with the mycelium growth rate method according the reported procedure [27]. Drazoxolon was co-tested as positive control. Every tested compound was dissolved in 0.5 mL DMSO and mixed with PSA (potato sucrose agar) medium (45 mL). The final concentration was 100 μg/mL. Meanwhile, 0.5 mL DMSO in 45 mL PSA medium was used as the control experiment. The medium was poured into three 9 cm petri plates uniformly, cooled, and solidified. The fungi were inoculated to the center of the medium. Then the treatments were cultured at 25 ± 1 °C for 3–5 days in the dark. The diameters of the fungal colonies were measured to calculate the growth inhibition rate when the Petri dishes had been covered two-thirds by the fungal colonies in the control treatment.

## 4. Conclusions

In this paper, a convenient synthesis of novel bioactive heterocycle compounds, phenylpyrrol-substituted tetramic acid derivatives bearing carbonates, was reported. The structures were well supported by spectroscopic data and single crystal X-ray diffraction analysis. The antifungal activity test indicated that these compounds showed obvious antifungal activities.

## Figures and Tables

**Table 1:** The crystal and experimental data of compound **6b**.

Crystal Data	
C_20_H_19_ClN_2_O_7_	α = 90°
*Mr* = 434.82	β = 95.588 (3)°
Monoclinic, *P*2_1_/*n*	γ = 90°
*a* = 10.0440 (9) Å	*V* = 2052.7 (4) Å^3^
*b* = 17.0692 (18) Å	*Z* = 4
*c* = 12.0304 (12) Å	μ = 0.231 mm^−1^
*F* (000) = 904	Crystal size (mm^3^): 0.38 × 0.42 × 0.45
**Data Collection**	
*T*_min_ = 0.903, *T*_max_ = 0.917	3078 observed reflections with I > 2σ(I)
19765 measured reflections	*R*_int_ = 0.030
4678 independent reflections	*θ*_max_ = 27.6°
**Refinement**	
*R*[*F*^2^ > 2σ(*F*^2^)] = 0.0596	*wR*(*F*^2^) = 0.1984
*S* = 1.02	Δρ_max_ = 0.53 e·Å^−3^, Δρ_min_ = −0.55 e·Å^−3^
4678 reflections	*w* [*P* = (*F_o_*^2^ + 2*F_c_*^2^)/3] = 1/[σ^2^(*F_o_*^2^) + (0.1106*P*)^2^ + 0.7217*P*]
293 parameters	Max. and Av. Shift/Error: 0.00, 0.00

**Table 2:** Selected geometric parameters of compound **6b** (Å).

N1-C1	1.375(3)	C4-C14	1.451(3)
C1-C2	1.348(3)	C14-C17	1.328(3)
C2-C3	1.402(3)	O4-C17	1.369(3)
C3-C4	1.356(3)	O4-C18	1.332(4)
N1-C4	1.398(3)	O5-C18	1.159(5)
N1-C8	1.428(3)	O6-C18	1.289(5)
O1-C1	1.373(3)	C19-C20	1.429(15)
O1-C5	1.356(3)	C19′-C20′	1.36(2)
O2-C5	1.176(3)	Cl1-C10	1.733(3)
O3-C5	1.301(3)		

**Table 3:** Hydrogen bonding data for compound **6b** (Å, °).

D-H···A	d(D-H)	d(H···A)	d(D···A)	∠(DHA)
C9-H9···O1	0.9300	2.5500	2.886(3)	101.00
C19-H19A···O5	0.9700	2.4300	2.772(12)	100.00
C16-H16B···O5	0.9700	2.4100	2.824(5)	105.00
N2-H2A···O7 ^a^	0.8600	2.0600	2.881(3)	159.00
C16-H16A···O2 ^b^	0.9700	2.5200	3.198(3)	127.00

Symmetry code for compound **6b**: ^a^ 3 − x, −y, 1 − z; ^b^ 5/2 − x, 1/2 + y, 1/2 − z.

**Table 4:** Percentage inhibition of compounds **6**, **4a**, and **5a** against three test fungi.

Compd.	*R*^1^	*R*^2^	Inhibition Rate (%) at 100 μg/mL		
			*F. graminearaum*	*B. cinerea*	*R. solani*
**6a**	3-Cl	Me	14.9 ± 2.8	27.1 ± 1.0	15.4 ± 3.6
**6b**	3-Cl	Et	27.4 ± 1.0	42.0 ± 3.5	35.0 ± 7.8
**6c**	3-Cl	*n*-Pr	47.4 ± 0.9	72.0 ± 1.1	73.2 ± 1.3
**6d**	3-Cl	*i*-Pr	45.1 ± 1.9	72.5 ± 1.9	78.7 ± 1.6
**6e**	3-Cl	*n*-Bu	46.5 ± 0.9	68.1 ± 1.1	67.7 ± 0.9
**6f**	3-Cl	*i*-Bu	48.8 ± 2.4	73.9 ± 1.1	74.8 ± 1.3
**6g**	3-Cl	Bn	31.6 ± 3.5	65.7 ± 1.0	60.2 ± 2.5
**6h**	H	*i*-Bu	51.8 ± 1.8	82.2 ± 3.0	66.7 ± 1.3
**6i**	4-F	*i*-Bu	53.7 ± 2.3	79.2 ± 1.6	70.6 ± 1.6
**6j**	2-Cl	*i*-Bu	48.6 ± 1.0	51.3 ± 2.5	60.7 ± 2.2
**6k**	4-Cl	*i*-Bu	39.4 ± 3.5	75.0 ± 1.6	71.4 ± 2.9
**6l**	4-Br	*i*-Bu	54.1 ± 3.0	70.3 ± 2.2	75.8 ± 3.0
**6m**	2-Me	*i*-Bu	52.8 ± 2.1	66.5 ± 3.8	69.4 ± 0.8
**6n**	3-Me	*i*-Bu	55.0 ± 2.4	77.1 ± 1.0	71.4 ± 2.2
**6o**	4-Me	*i*-Bu	46.3 ± 3.6	71.6 ± 2.1	73.4 ± 3.5
**6p**	3-OMe	*i*-Bu	60.6 ± 7.1	61.9 ± 1.0	69.8 ± 6.7
**6q**	3-CF_3_	*i*-Bu	40.4 ± 1.1	56.4 ± 2.1	63.5 ± 3.4
**4a**	H	—	1.8 ± 1.4	16.2 ± 2.3	0
**5a**	H	—	2.5 ± 1.8	14.3 ± 1.2	2.5 ± 1.0
Drazoxolon	—	—	75.1 ± 2.3	95.0 ± 1.5	94.9 ± 1.1

## Supplementary Materials

Supplementary materials can be accessed at: http://www.mdpi.com/1420-3049/21/3/355/s1.
